# ProjDIA: Fragment-Bin Projection Scoring and Calibrated Confidence for Direct Infusion DIA Proteomics

**DOI:** 10.1021/acsmeasuresciau.6c00119

**Published:** 2026-07-30

**Authors:** Herbi Yuliantoro, Peng Li

**Affiliations:** Department of Chemistry, 1687 Lehigh University, 6 East Packer Avenue, Bethlehem, Pennsylvania 18015, United States

**Keywords:** direct-infusion proteomics, data-independent acquisition, mass spectrometry, peptide identification, computational proteomics, label-free quantification

## Abstract

Liquid chromatography–mass spectrometry (LC-MS) is the most powerful tool for bottom-up proteomics workflows but limits throughput because separations are slow and experimentally complex. Direct infusion shotgun proteome analysis (DI-SPA) offers a faster alternative, but highly chimeric tandem mass spectra complicate peptide identification and quantification. Here, we present ProjDIA, a computational workflow for DI-SPA that combines fragment-bin projection scoring, confidence-guided two-pass mass-error recalibration, monotone q-value reporting, and guarded semisupervised rescoring. Across public HeLa DI-SPA benchmarks, ProjDIA increased peptide-level identifications relative to CsoDIAq and showed condition-dependent gains relative to CHIMERYS, with the clearest advantage observed in the sample-load series and at higher MS2 resolution. In species-entrapment analysis, ProjDIA showed that these gains are not at the expense of higher error rates. Finally, ProjDIA also supported label-free quantification and demonstrated the gains in peptide identification can lead to deeper coverage of proteins with significant fold changes under perturbation.

## Graphical abstract

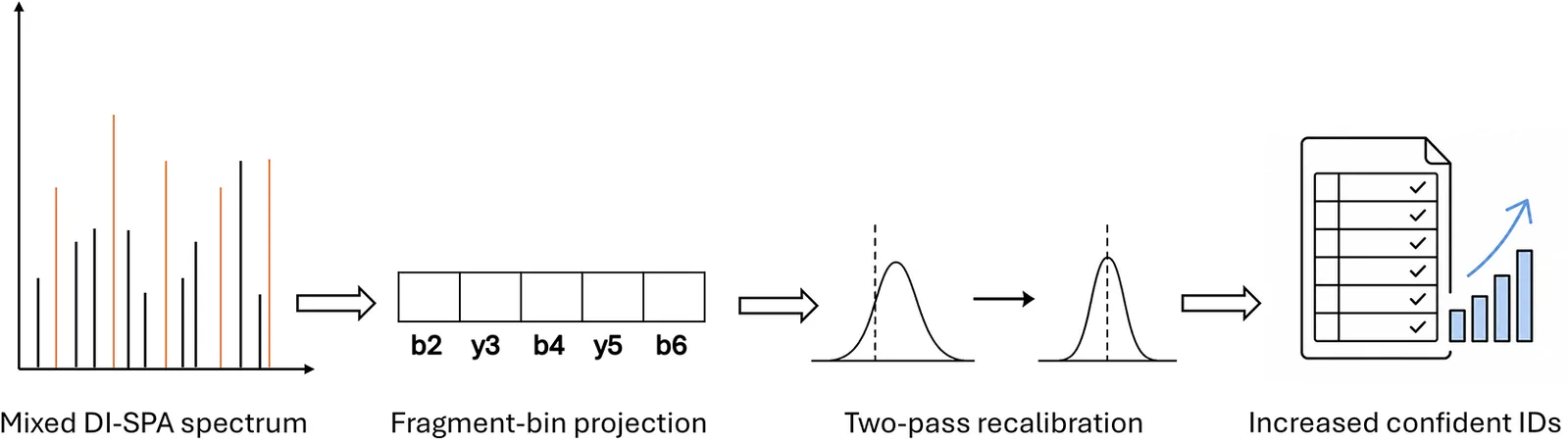

## Introduction

Bottom-up proteomics is a key technology for comprehensive protein analysis and has become indispensable in applications ranging from fundamental biology to translational and clinical research.^1^ To date, most bottom-up proteomics experiments are performed by liquid chromatography–mass spectrometry (LC–MS), largely because chromatographic separation reduces sample complexity and enables sensitive, deep, and reliable peptide identification.^2,3^ Despite these advantages, LC–MS imposes practical limitations on experiment throughput because LC separations and the needs of high-pressure fluidic systems add time, increase workflow complexity, and constrain the analysis of large sample series.^4^ Direct infusion shotgun proteome^5^ analysis (DI-SPA) represents an attractive alternative, as it removes chromatographic separation and can provide substantially faster and simpler analyses for high-throughput applications.^6–11^ However, in the absence of LC, DI-SPA suffers ion suppression and produces highly complex, mixed MS2 spectra, which still restrict proteome depth. Realizing the full promise of DI-SPA will therefore require further improvements across multiple fronts, including instrumentation, acquisition design, and computational methods.^12^

DI-SPA typically employs DIA acquisition because, without LC separation, many peptides are analyzed and fragmented simultaneously. Consequently, DI-SPA MS2 spectra are highly complex and strongly chimeric.^5,13,14^ In LC-DIA, many existing analysis tools exploit the chromatographic time dimension for peptide identification by tracking fragment ions that coelute with the same precursor. Therefore, widely used DIA software, including DIA-NN,^15^ DIA-Umpire,^16^ AlphaDIA,^17^ EncyclopeDIA,^18^ PECAN,^19^ and Spectronaut, is generally not applicable to DI-SPA data, where no elution profiles are present.

MSPLIT-DIA^20^ was an early and important step toward addressing this problem. It introduced a projected-spectrum approach for analyzing chimeric DIA spectra based solely on spectral information, without requiring chromatographic separation.^21^ Building on the principles established by MSPLIT-DIA, CsoDIAq^22^ was subsequently developed as an open-source workflow designed to improve DI-SPA data processing. CsoDIAq increased the practicality of DI-SPA analysis by introducing spectra pooling, the match count and cosine (MaCC) score, and fragment mass-tolerance correction, leading to improved speed and sensitivity. Later studies showed that this approach enabled the identification of up to ∼2000 proteins and quantification of ∼1000 proteins within minutes^13^. More recently, RE-FIGS extended DI-SPA analysis to repeated-acquisition experiments by combining cycle-aware feature extraction, machine-learning-based scoring, and featured-ion-based quantification to improve sensitivity.^23^ Commercial software, CHIMERYS,^24^ also demonstrates the ability to analyze highly complex chimeric spectra from DI-SPA experiments.

Despite this progress, software for DI-SPA remains limited, and several issues still affect the sensitivity, robustness, and confidence estimation of DI-SPA data processing. First, library-based DI-SPA analysis workflows rely on spectrum-to-library matching in which only query peaks that fall within tolerance of expected library fragments contribute to the score. This matched-only strategy suppresses unrelated signals, but it does not directly penalize missing expected fragments, so candidates supported by only a few coincidental matches can still rank well.^20,22^ Second, fragment-mass recalibration can be distorted when fitted from broad sets of preliminary matches that include false positives, and confidence is often reported mainly as filtered result lists rather than as calibrated per-match q-values.^25^ Third, while RE-FIGS has shown that machine-learning-based scoring can improve direct-infusion data analysis, it depends on repeated-acquisition-specific features, reflecting a trade-off between sensitivity and the rapid-throughput goal of DI-SPA.

Here we introduce ProjDIA (Figure 1), a DI-SPA workflow that addresses these issues by combining fragment-bin projection scoring, confidence-guided two-pass mass-error recalibration based on robust statistics, and monotone q-value reporting with guarded semisupervised rescoring, thereby improving identification sensitivity and confidence calibration in DI-SPA data. To assess the performance of ProjDIA, we benchmarked it against state-of-the-art DI-SPA software, CsoDIAq and CHIMERYS, on large DI-SPA data sets to evaluate identification and quantification performance across many replicates. We performed module ablations to quantify the contribution of key workflow components. We examined the peptide identification performance under common DI-SPA acquisition variables, including MS2 resolution and sample load. To evaluate peptide discovery quality, we used two complementary negative-control strategies: decoys and species entrapment.^26^ We further evaluated label-free quantification^27^ on a defined three-species mixture to demonstrate quantification performance in DI-SPA data. Finally, we examined whether these methodological improvements could translate into better preservation of biologically meaningful patterns in multifactorial perturbation data, supporting the use of DI-SPA for rapid screening applications.

**Figure 1:**
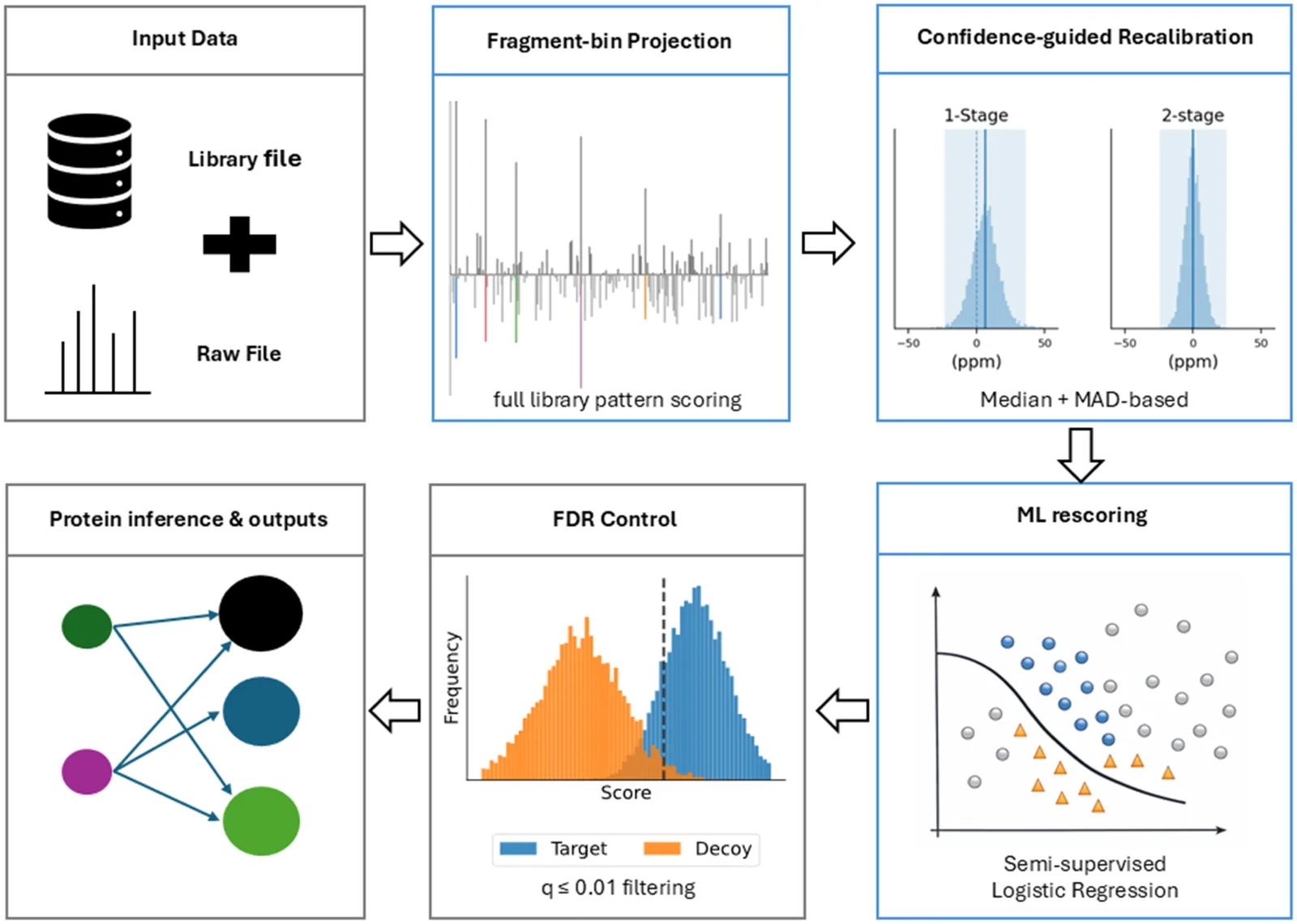
ProjDIA workflow schematic. Pipeline steps include windowed spectral matching, fragment-bin projection scoring, two-pass fragment mass-error recalibration, ML rescoring, and reporting of final identifications with monotone q-values at the chosen FDR cutoff.

## Results

### Workflow Overview

ProjDIA processes mzXML^28^ files against a spectral library containing targets and appended decoys,^29,30^ analyzing each run by DIA isolation window. MS2 scans are grouped by isolation-window center *m*/*z*, window width, and FAIMS compensation voltage (when present), so scans from different windows or FAIMS planes are not combined.^31^ Within each group, ProjDIA pools library fragment peaks for candidate precursors in the same window and query peaks in small scan chunks while retaining scan identifiers, which reduces redundant comparisons while preserving scan-specific matches, inspired by CsoDIAq and MS-Fragger.^32^ Candidate peptide-spectrum matches are formed by fragment pairing within a user-defined ppm tolerance and are required to meet a minimum shared-fragment threshold before scoring. Matches are scored using a projected representation in which observed query intensities are mapped onto the library’s full fragment list, with unmatched expected fragments contributing zeros. A max-per-bin rule prevents multiple query peaks from inflating a single library fragment position. Candidates are ranked using a MaCC-style score^22^ derived from projected cosine similarity and the number of shared fragment bins. ProjDIA then estimates target-decoy confidence and computes monotone q-values, which are used to drive a two-pass mass-error recalibration: high-confidence targets from pass 1 (default *q* ≤ 0.01) are used to fit the run-specific mass-error center and spread (median and MAD), and matches are filtered and rescored in pass 2 using the recalibrated tolerance. Finally, ProjDIA applies semisupervised logistic-regression rescoring using match-quality features, with safeguards for small data sets and optional scan-grouped cross-validation. The workflow outputs peptide spectrum matches (PSM), where each PSM denotes a scored match between one experimental DI-SPA MS2 scan and one library precursor candidate, peptide, and protein tables, and performs protein inference using an IDPicker-style parsimony procedure.^33,34^ For context, Table S1 summarizes the practical and algorithmic differences among CsoDIAq, RE-FIGS, and ProjDIA, and Table S2 lists the source data set, accession number, and corresponding analysis section for each benchmark and downstream analysis.

### Determination of the Contribution of Each Pipeline Module to the Peptide Identification

In ProjDIA, the key improvements lie in 3 components that are fragment-bin projection scoring, two-pass mass-error recalibration, and machine-learning rescoring. To quantify the contribution of each of these components, we performed a module ablation study on DI-SPA data acquired at multiple MS2 resolutions (30k, 45k, 60k, 120k, and 240k).^13^ We compared the full pipeline to runs in which one module was disabled at a time: fragment-bin projection scoring (“No projection” or matched-only scoring), two-pass mass-error recalibration (“No recalibration”), or machine-learning rescoring (“No ML rescoring”). Identification yields were evaluated at a fixed confidence threshold of *q* ≤ 0.01 (Figure S1).

Under all resolutions, the full pipeline produced the highest peptide and protein yields, showing that the modules are complementary rather than redundant. The weight of each module depends on the resolution setting. Disabling projection scoring caused a consistent loss in identifications and was most pronounced at low resolution, where fragment peaks are less well resolved and more likely to be overlapped or convoluted, increasing ambiguity in assigning observed signal to specific library fragments. Disabling mass-error recalibration had a modest effect at 30k but caused progressively larger losses at higher resolutions, consistent with small run-specific ppm shifts becoming limiting when fragment peaks are narrower. ML rescoring provided a smaller but reproducible gain, improving separation near the decision boundary without replacing the core scoring model.

The retention heatmaps (Figure S2) quantify overlap with the full pipeline and highlight these resolution-dependent effects. For peptides, removing projection retained 70.7% of full IDs at 30k and 78.1% at 240k, whereas removing recalibration retained 90.4% at 30k but only 53.9% at 240k. Removing ML rescoring retained 92.0–97.8% (and 100% at 30k), indicating rescoring refines borderline calls. Similar patterns were observed for PSMs and proteins. In conclusion, the yield trends and retention analysis show that projection scoring, confidence-guided recalibration, and ML rescoring make distinct, nonredundant contributions to the final confident identifications.

### Peptide Identification under Different Experimental Conditions

We compared the performance of peptide identification of ProjDIA with state-of-the-art DI-SPA software including the open-source CsoDIAq and the proprietary CHIMERYS. CHIMERYS was included as a practical external comparator because its original publication reports native support for DI-SPA analysis and includes DI-SPA benchmarking against CsoDIAq.^24^ However, the comparison is interpreted as a workflow-level benchmark rather than a direct one-to-one algorithmic comparison, because ProjDIA and CsoDIAq are spectral-library-based DI-SPA workflows, whereas CHIMERYS is a spectrum-centric, library-free workflow with different search construction, scoring, FDR implementation, and output structure. We analyzed a published HeLa digest standard data set^13^ spanning MS2 resolutions from 30k to 240k and sample loads from 0.125 to 1 μg. Identifications were reported at *q* ≤ 0.01 for each workflow. Because these tools differ in scoring geometry, calibration strategy and search design, each software was run with its recommended settings rather than force-matched non-native parameters; for the library-based software (ProjDIA and CsoDIAq), the same library was used to focus the benchmark on workflow design rather than library composition.

Across the MS2 resolution series, all three tools benefited from higher resolution, but the gain was strongest for ProjDIA, particularly at the peptide level (Figure 2a, b). For PSMs, CHIMERYS gave the highest mean counts at 30k–60k resolution, suggesting that it may be better at recovering spectrum-level matches from lower-resolution MS2 data in which fragment peaks are broader, less resolved, and more frequently convoluted. However, this advantage was weaker at the peptide level, indicating that the additional CHIMERYS PSMs did not translate proportionally into broader unique peptide recovery. Because PSMs and unique peptides represent different levels of summarization, their counts are not expected to scale linearly: multiple spectrum-level matches can support the same peptide sequence, so additional PSMs can reflect either broader peptide recovery or additional redundant support for peptides already identified. Table S3 summarizes PSM-to-peptide collapse metrics for the Figure 2 benchmarks. In the sample-load series, ProjDIA produced more unique peptides than CHIMERYS at every tested load while maintaining a lower PSMs-per-unique-peptide ratio (1.39–1.42 for ProjDIA versus 1.60–1.64 for CHIMERYS), indicating that ProjDIA’s spectrum-level evidence translated more efficiently into unique peptide recovery under these tested loading conditions. CsoDIAq showed similar or slightly lower collapse ratios in some settings but reported fewer total PSMs and unique peptides, so the collapse metrics should be interpreted together with identification yield rather than as a standalone performance measure.

**Figure 2:**
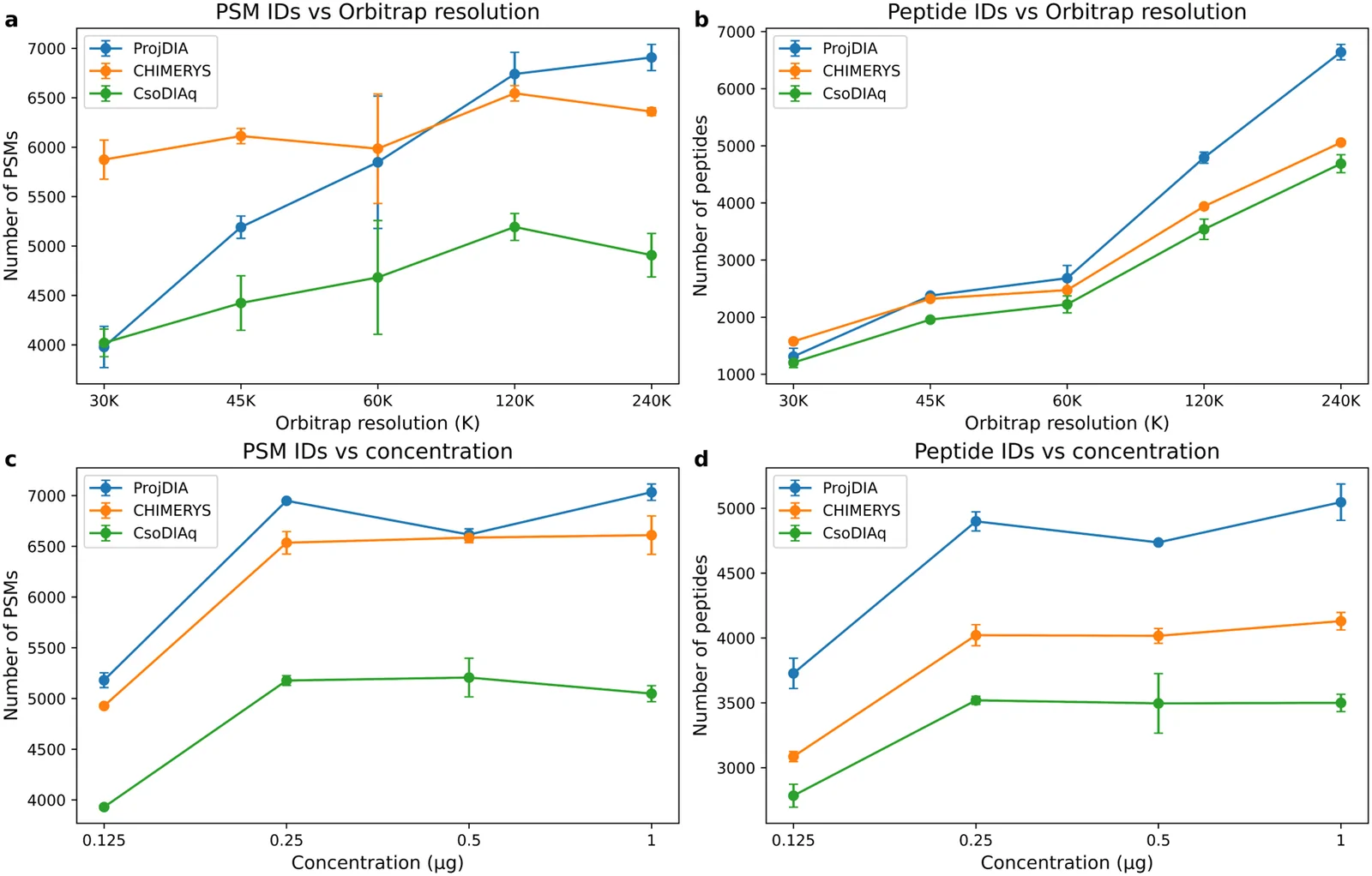
Benchmarking identification performance across MS2 resolution and sample load using a HeLa DI-SPA standard. Numbers of confident identifications reported by ProjDIA, CHIMERYS, and CsoDIAq from common DI-SPA acquisition settings. (a,b) PSMs (a) and unique peptides (b) at *q* ≤ 0.01 as a function of Orbitrap MS2 resolution (30K–240 K). (c,d) PSMs (c) and unique peptides (d) at *q* ≤ 0.01 as a function of sample load (0.125–1 μg) at 120 K acquisition settings. Points show the mean across the public replicate acquisitions included in the benchmark summary files, and error bars show the standard deviation across replicates. Replicate numbers were *n* = 2 for the sample-load conditions and for the 30K, 45K, and 60K resolution conditions, and *n* = 3 for the 120 and 240 K resolution conditions. Paired replicate-level differences, 95% confidence intervals, and paired-test results are reported in Table S4.

ProjDIA increased more steeply with resolution and became the top-performing method at 120k and 240k, reaching 6,740 and 6,908 mean PSMs, respectively, compared with 6,545 and 6,360 for CHIMERYS and 5,193 and 4,907 for CsoDIAq. The peptide-level trend favored ProjDIA more clearly, after being nearly identical to CHIMERYS at 45k (2,377 versus 2,322 mean peptides), ProjDIA exceeded both CHIMERYS and CsoDIAq from 60k onward, reaching 4,792 peptides at 120k and 6,641 at 240k, compared with 3,939 and 5,057 for CHIMERYS and 3,539 and 4,687 for CsoDIAq. Together, these results show that ProjDIA reported the greatest benefit from higher-resolution MS2 data, with its clearest advantage emerging for peptide identification at 120k–240k.

Moreover, ProjDIA produced the highest mean yield of confident identifications for both PSMs and peptides at every tested sample amount (Figure 2c, d). At the lowest load (0.125 μg), ProjDIA identified 5,181 PSMs and 3,728 peptides, compared with 4,927 and 3,087 for CHIMERYS and 3,930 and 2,785 for CsoDIAq. At the highest load (1 μg), ProjDIA identified 7,034 PSMs and 5,047 peptides, exceeding 6,610 and 4,130 for CHIMERYS and 5,048 and 3,500 for CsoDIAq. Identification yields increased from 0.125 to 0.25 μg and then approached a plateau, but ProjDIA maintained an advantage across the full loading range, particularly at the peptide level. At the lowest and highest tested loads, ProjDIA increased peptide identifications by 20.8% and 22.2% relative to CHIMERYS, and by 33.9% and 44.2% relative to CsoDIAq, respectively. Because each public replicate acquisition was processed by each workflow, workflow differences were evaluated using paired replicate-level comparisons within each acquisition condition. The corresponding mean paired differences, relative differences, 95% confidence intervals, and paired-test results are reported in Table S4.

To further evaluate the fragment-level evidence supporting accepted assignments, we analyzed representative matched subsets from the resolution and sample-load benchmarks (Figure S3 and Tables S5–S6). In these subsets, ProjDIA accepted more target PSMs than CsoDIAq, whereas CsoDIAq showed a higher median number of matched fragments and fewer missing expected fragments per accepted PSM. ProjDIA accepted PSMs were still supported by multiple matched fragments, and fragment-sharing ambiguity was low: 97.86% and 98.04% of matched observed peaks were compatible with only one accepted peptide candidate in the 0.5 μg/240k and 1 μg/120k subsets, respectively. These results indicate that ProjDIA broadens peptide recovery while retaining multifragment support and limited fragment sharing within isolation windows. Together, the resolution and sample-load benchmarks show that ProjDIA’s identification gains, particularly at the peptide level, persist across representative DI-SPA acquisition settings.

### Stability in Large Replicate Studies, Repeated Acquisition Data, and Runtime Analysis

We next evaluated performance in a replicate-heavy DI-SPA data set using 100 consecutive acquisitions of the same MCF-7 sample data from Orbitrap Lumos.^5^ For each run, we reported confident identifications at a matched threshold (*q* ≤ 0.01) and compared ProjDIA to CsoDIAq and CHIMERYS (Figure 3a, b). Among 100 replicate DI-SPA runs, ProjDIA consistently delivered the highest identification yields. For PSMs, ProjDIA achieved a median of 7,839.5 (vs 5,677.5 for CHIMERYS and 3,378.0 for CsoDIAq) while showing the lowest run-to-run variability (CV 5.87%, vs 7.73% and 6.71%, respectively). Importantly, the higher yield of ProjDIA was not accompanied by greater instability across runs. For PSMs, the 5–95% range was 1,507.3 for ProjDIA, essentially identical to 1,505.9 for CHIMERYS. ProjDIA also showed only one outlier run, compared with four for CHIMERYS and three for CsoDIAq, indicating that its improved yield was sustained across the replicate series rather than driven by a small number of exceptional runs. At the peptide level, ProjDIA showed the same overall pattern, with a median of 4,215 peptides per run, compared with 3,483 for CHIMERYS and 2,888 for CsoDIAq, together with moderate variability (CV 6.76%) and only one outlier.

**Figure 3:**
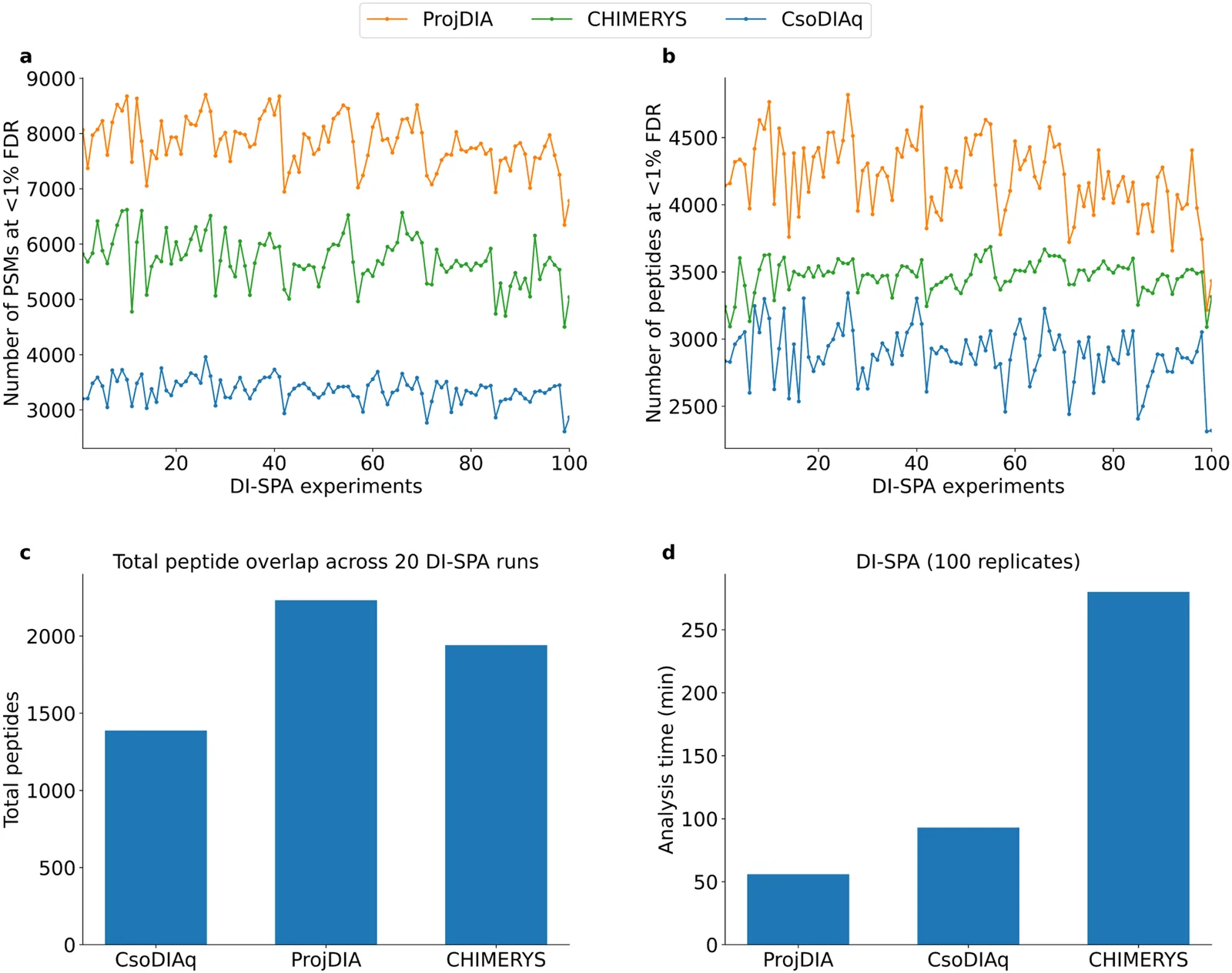
Stability and throughput from 100 repeated DI-SPA acquisitions of an MCF-7 sample. (a,b) Numbers of confident identifications at *q* ≤ 0.01 for each of 100 consecutive DI-SPA runs, shown for PSMs (a) and peptides (b) for ProjDIA, CsoDIAq, and CHIMERYS, enabling comparison of yield and run-to-run stability within a large replicate series. (c) Reproducible peptide core in a representative matched 20-run subset, defined as peptides identified at *q* ≤ 0.01 in all 20 selected runs within each workflow. (d) Wall-clock analysis time for the 100-run MCF-7 data set. ProjDIA and CsoDIAq were run on the same local Windows workstation and are directly comparable. CHIMERYS was evaluated through the cloud-based MSAID Platform trial; the reported time corresponds to the logged analysis-stage runtime after data submission and is shown only as cloud-platform operational context, not as a direct local runtime comparison.

To further assess reproducible peptide recovery across repeated measurements, we selected a representative matched 20-run subset and counted peptides that were consistently identified in all 20 runs within each workflow (Figure 3c). This stringent run-consistency analysis evaluates within-workflow reproducibility rather than cumulative peptide discovery. Using this criterion, ProjDIA recovered the largest reproducible peptide set, with 2,232 peptides identified in all 20 selected runs, compared with 1,941 for CHIMERYS and 1,388 for CsoDIAq. Thus, ProjDIA’s higher per-run yield was accompanied by a larger set of repeatedly detected peptides across the matched replicate subset.

We next asked whether the peptides recovered by different workflows represented mostly overlapping or largely workflow-specific sequence sets. For this analysis, we used a separate pooled-union comparison in which peptide lists from the selected runs were pooled within each workflow, deduplicated, and then compared across workflows (Figure S4). Because ProjDIA and CsoDIAq are library-based workflows whereas CHIMERYS is library-free, this comparison was performed at the normalized peptide-sequence level rather than as a direct PSM-level reassignment analysis. The pooled-union analysis showed substantial overlap among workflows, with 3,980 peptide sequences shared by all three workflows in the 0.5 μg, 240k subset, 2,977 in the 1 μg, 120k subset, and 3,421 in the pooled MCF-7 20-run subset. ProjDIA also recovered additional workflow-specific peptides in each setting, indicating that its gains reflect both shared peptide recovery and additional peptide identifications rather than a completely unrelated identification set.

Large replicate collections also place practical pressure on analysis throughput. We therefore first compared the two workflows executed on the same local workstation. ProjDIA completed the 100-run MCF-7 batch in 56 min, compared with 93 min for CsoDIAq (Figure 3d), corresponding to approximately 34 s per run for ProjDIA and 56 s per run for CsoDIAq. Thus, under matched local workstation conditions, ProjDIA was approximately 1.7-fold faster than CsoDIAq. CHIMERYS was evaluated through the cloud-based MSAID Platform trial, and the CHIMERYS time shown in the workflow log corresponded to the analysis stage after data submission rather than local CPU execution. Because the CHIMERYS runtime was obtained on different infrastructure with platform-specific resource allocation and account settings, we report it only as cloud-platform operational context and do not use it to infer direct algorithmic speed relative to ProjDIA or CsoDIAq. These results show that ProjDIA improves both identification robustness across DI-SPA acquisitions and the computational throughput needed to analyze large replicate studies.

Furthermore, to confirm that the stability trends were not specific to the 100-run MCF-7 collection, we analyzed a different type of acquisition (repeated-acquisition) data set from the RE-FIGS study,^23^ consisting of 12 repeated DI-SPA acquisitions of a HeLa digest standard. At *q* ≤ 0.01, ProjDIA reported consistently higher PSM, peptide and protein identifications across all 12 runs and showed substantially lower run-to-run variability than CsoDIAq, with coefficients of variation of 1.4% vs 10.4% (PSMs), 2.1% vs 13.6% (peptides), and 2.1% vs 17.9% (proteins) (Figure S5). Together with the 100-run MCF-7 analysis, these results support stable ProjDIA performance across replicate-heavy DI-SPA designs and across data sets acquired under different experimental settings.

### Entrapment-Based Evaluation of Identification Confidence and Rescoring Safeguards

Species-entrapment analysis was used as an empirical check of accepted peptide identifications, using two complementary entrapment designs. In the species-mixture analyses, human/sample entries were treated as target entries, whereas *E. coli* and yeast entries were used as absent-species entrapment entries; because *E. coli* and yeast peptides are not expected in the HeLa sample, accepted entrapment identifications provide a direct indication of false matching among accepted results. In the ProjDIA holdout control, a paired reference-entrapment design was used instead, in which each target peptide is matched to one entrapment (TRAP) counterpart (see below). For each workflow, outputs were filtered at *q* ≤ 0.01, decoys were excluded and accepted nondecoy peptides were classified as target or entrapment. The observed entrapment rate was calculated as E/(T+E), where E is the number of accepted entrapment peptides and T is the number of accepted target peptides. This observed entrapment rate is reported separately from the entrapment-based false discovery proportion (FDP) estimate, E­(1 + 1/r)/(T+E), where r = N_entrapment_/N_target_ accounts for the relative sizes of the entrapment and target search spaces.

In the ten-run spectral-library species-entrapment analysis, ProjDIA and CsoDIAq were compared using the same target/entrapment spectral-library design. After excluding 19 shared target/entrapment peptide sequences and artificial decoys, the searchable peptide space contained 16,623 target peptides and 27,171 entrapment peptides, corresponding to r = 1.634543 and an adjustment multiplier of 1 + 1/r = 1.611792. At *q* ≤ 0.01, ProjDIA reported 2,712 accepted target peptides and 23 accepted entrapment peptides, corresponding to an observed entrapment rate of 0.8410% and an entrapment-based FDP estimate of 1.3554%. CsoDIAq reported 1,134 accepted target peptides and 9 accepted entrapment peptides, corresponding to an observed entrapment rate of 0.7874% and an entrapment-based FDP estimate of 1.2691% (Figure 4A; Table S7). Thus, in this direct spectral-library comparison, ProjDIA recovered approximately 2.4-fold more accepted target peptides than CsoDIAq while maintaining a comparable observed entrapment rate and a comparable entrapment-based FDP estimate.

**Figure 4:**
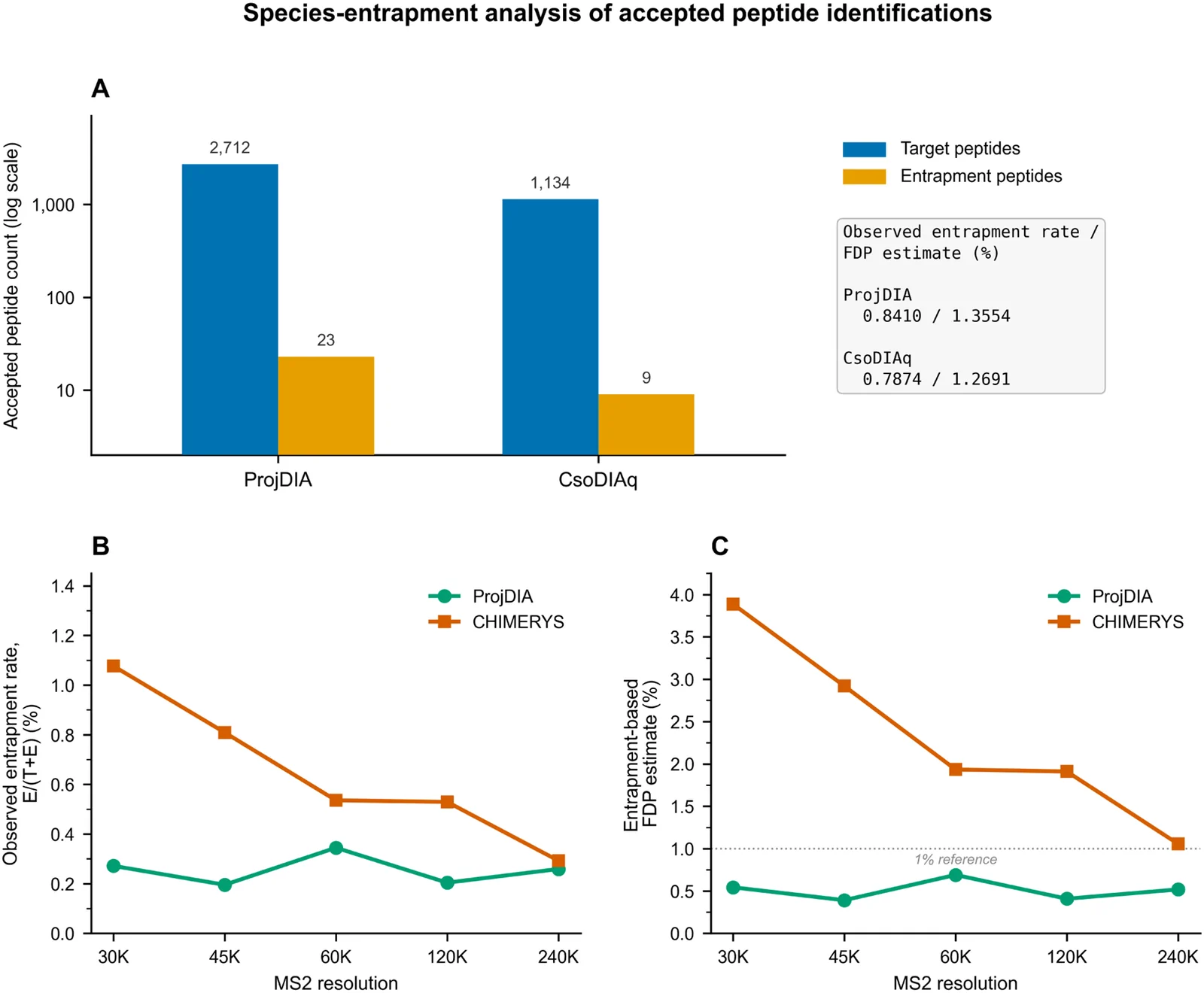
Species-entrapment analysis of accepted peptide identifications. (A) Ten-run spectral-library species-entrapment analysis comparing ProjDIA and CsoDIAq at *q* ≤ 0.01. Bars show accepted target and entrapment peptide identifications on a log scale. Labels report the observed entrapment rate, E/(T+E), and the entrapment-based false discovery proportion (FDP) estimate, E­(1 + 1/r)/(T+E), where T is the number of accepted target peptides, E is the number of accepted entrapment peptides, and r = N_entrapment_/N_target_. (B) Observed peptide-level entrapment rates across MS2 resolutions for the ProjDIA entrapment holdout control and CHIMERYS postsearch species assignment. In the ProjDIA control, entrapment-origin entries remained searchable and reportable but were held out from mass-error recalibration and rescoring model fitting. For CHIMERYS, accepted peptide sequences were classified after searching by assignment to in-silico human, *E. coli*, and yeast proteomes, with shared target/entrapment sequences excluded. The dotted line, if shown, is a 1% visual reference only and is not used as a formal FDP threshold. (C) Corresponding database-size-adjusted entrapment-based FDP estimates. CHIMERYS values represent a postsearch mapping-based diagnostic and do not constitute an audit of its proprietary internal FDR estimation.

We also performed a ProjDIA entrapment holdout control to ensure that entrapment-origin entries remained external to confidence-model fitting. This control used a paired reference-entrapment library in which every target peptide had exactly one entrapment (TRAP) counterpart, giving equal target and entrapment search-space sizes (N_target_ = N_entrapment_ = 86,725 unique peptides; r = 1, so the entrapment-based FDP estimate reduces to 2E/(T+E)). Entrapment-origin entries remained searchable and reportable but were held out from mass-error recalibration fitting and from high-confidence target selection during semisupervised rescoring; decoys generated from entrapment-origin entries were likewise held out from rescoring model fitting, whereas target-derived decoys remained available as decoy examples. Across the MS2-resolution series, observed peptide-level entrapment rates at *q* ≤ 0.01 remained low, ranging from 0.1953% to 0.3447%, with corresponding entrapment-based FDP estimates of 0.3905% to 0.6894% (Figure 4B). These results indicate that the low observed entrapment rates did not depend on using entrapment-origin entries during recalibration or rescoring.

For comparison with the library-free CHIMERYS workflow, accepted peptide sequences were classified after searching by postsearch species assignment to in-silico human, *E. coli*, and yeast proteomes. The CHIMERYS search space was constructed by in-silico Trypsin/P digestion of the combined human, *E. coli*, and yeast proteomes and, after excluding 2,214 shared sequences, contained 2,102,616 target and 805,580 entrapment peptides (r = 0.383132; 1 + 1/r = 3.610065). Raw observed entrapment rates decreased from 1.0766% at 30K to 0.2925% at 240 K (Figure 4B). After adjustment for the unequal search-space sizes, the corresponding entrapment-based FDP estimates ranged from 3.8867% to 1.0560% (Figure 4C). Under the tested conditions, the ProjDIA holdout control produced lower raw entrapment rates and lower adjusted FDP estimates. However, because ProjDIA and CHIMERYS differ in search design, search-space construction, and confidence estimation, these results are interpreted as workflow-level external entrapment diagnostics rather than a direct comparison of internal FDR calibration or identification performance. CHIMERYS values specifically represent a postsearch mapping-based diagnostic and do not audit its proprietary internal FDR estimation.

We further evaluated whether recalibration or semisupervised rescoring could introduce in-sample optimism. The recalibration/rescoring safeguards are summarized in Table S8. In the entrapment holdout-control series, final q-values were computed either from base scores or from scan-grouped held-out fold scores. ML rescoring was skipped at 30K, 45K, and 60K because excluding entrapment-derived decoys reduced the eligible-decoy count below the rescoring guard, whereas 120 and 240 K used held-out fold logit scores. In the controlled 120 K comparison, excluding entrapment-origin entries from recalibration and rescoring model fitting did not increase the observed peptide-level entrapment rate or the entrapment-based FDP estimate. These results indicate that the low entrapment signal was not dependent on reporting in-sample ML training scores.

The analyses above evaluate accepted peptide identifications. Because protein groups were inferred from peptides accepted at *q* ≤ 0.01 rather than controlled by an independent protein-level FDR procedure, we also evaluated protein-level entrapment behavior separately (Table S9). In the ten-run species-entrapment analysis, the observed protein-level entrapment fraction was 2.09% (22 entrapment among 1,052 protein groups). This value is an observed diagnostic, not a formal protein-level FDR estimate. The signal was mainly driven by single-peptide protein groups: 21 of the 22 entrapment protein groups were supported by one peptide. Requiring ≥ 2 supporting peptides reduced the observed protein-level entrapment fraction to 0.15% using ≥ 2 peptides total or 0.00% using ≥ 2 unique peptides. Therefore, protein-level counts are reported as supportive peptide-level-filtered protein-group summaries.

We next asked whether rescoring improves discrimination between true and false matches. Mirrored histograms show that the rescored mlScore separates sample targets from both decoys and species-entrapment matches, with false matches concentrated at lower scores (Figure S6a,b). The q-value distributions show that decoy and entrapment identifications are enriched at higher q-values and largely depleted below *q* ≤ 0.01 (Figure S6c,d). These internal diagnostics support that the score-to-q mapping produces a clear separation, complementing the entrapment-based evaluation shown in Figure 4.

### Label-Free Quantification from DI-SPA Using ProjDIA

To assess ProjDIA’s suitability for label-free quantification (LFQ) in highly multiplexed DI-SPA data, we analyzed a defined three-species mixture and a HeLa dilution series. ProjDIA reports peptide- and protein-level intensity tables after identification filtering, enabling ratio-based comparisons between sample conditions.

In the three-species mixture, ProjDIA recovered species-specific quantitative patterns across a broad intensity range. At both the protein and peptide levels, expected fold changes were defined by the mixture design (yeast: + 1, human: 0, and *E. coli*: −2 on the log2 scale), plotted as horizontal reference lines, and summarized using abundance-stratified heatmaps across species and intensity bins (Figure S7). To focus on stable quantitative features, we retained features quantified in ≥ 4 replicates per condition with CV < 0.20 in both A and B. Using these filtered features, unsupervised analysis of quantified peptides showed clear global structure: PCA separated yeast, human, and *E. coli* into distinct clusters, with PC1 explaining 92.3% of the variance and PC2 explaining 7.7% (Figure 5a). Quantitative accuracy was summarized by the distribution of log2 ratio error (observed – expected), with median error (MAD) values of +0.211 (0.118) for yeast, + 0.139 (0.077) for human, and −0.067 (0.148) for *E. coli* (Figure 5b). These values indicate that ProjDIA recovered the expected species-specific ratio structure, although modest systematic LFQ offsets remained.

**Figure 5:**
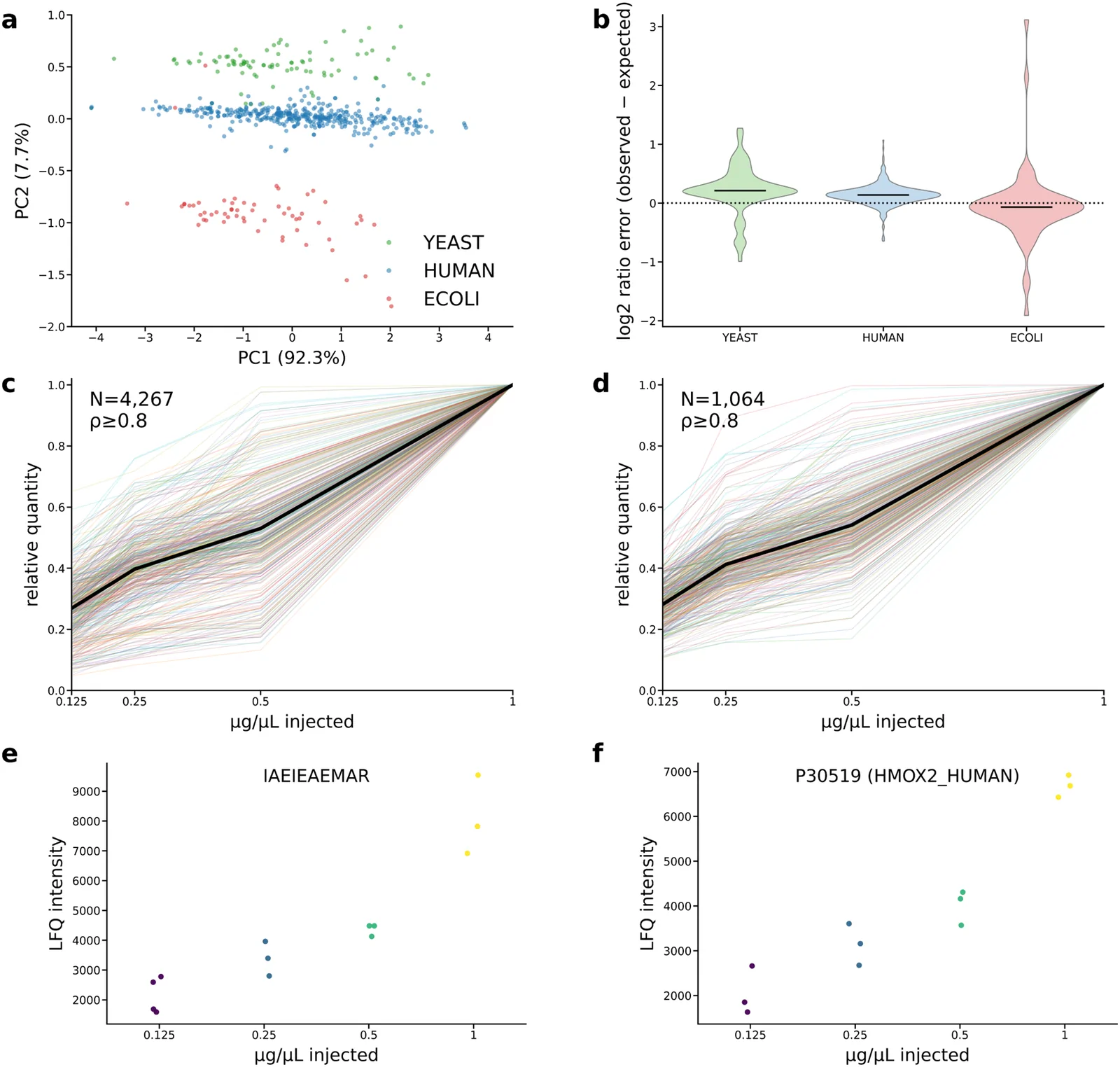
Label-free quantification from DI-SPA using ProjDIA. (a) PCA of quantified peptides from a defined three-species mixture shows clear separation by species (yeast, human, *E. coli*, variance explained shown on axes), indicating coherent quantitative structure. (b) Log2 ratio error (observed – expected) for quantified features summarizes quantitative accuracy across species. (c–d) HeLa dilution series (0.125–1 μg/μL injected) showing normalized peptide-level (c) and protein-level (d) LFQ profiles for features with positive concentration slope. The bold line shows the median response. Insets report the number of features with strong response (Spearman ρ ≥ 0.8). (e–f) Representative examples of a peptide (e) and protein (f) illustrate replicate-level LFQ intensities increasing with injected amount.

To determine whether these offsets were specific to ProjDIA, we compared ProjDIA with CsoDIAq using the same raw files, spectral library, mass tolerance, MaxLFQ quantification, and downstream filters (Table S10). CsoDIAq showed median peptide-level log2­(A/B) ratio errors of −0.200 for yeast, + 0.109 for human, and +0.489 for *E. coli*. The small positive offset in the human 1:1 channel was observed for both workflows, consistent with a shared sample-level or normalization component. ProjDIA preserved the expected yeast-to-*E. coli* log2 separation more closely than CsoDIAq at both the peptide and protein levels, indicating less ratio compression under these matched conditions.

Finally, concentration-dependent LFQ was evaluated using a HeLa dilution series (0.125–1 μg/μL injected). For each peptide and protein, LFQ intensities were averaged within each concentration and monotonicity was assessed only for features quantified at all tested concentrations. Thousands of features increased monotonically with injected amount: 4,267 peptides and 1,064 proteins showed a positive slope and strong monotonic response (Spearman ρ ≥ 0.8) (Figure 5c–d). Representative peptide and protein examples showed consistent replicate intensities and smooth increases with concentration (Figure 5e–f). These results demonstrate that ProjDIA supports reproducible LFQ from DI-SPA, capturing both expected mixture ratios and concentration-dependent quantitative behavior.

### ProjDIA Supports Recovery of Toxin-Response Patterns in Multifactorial Screening

Having established that ProjDIA supports reproducible label-free quantification, we next asked whether these analytical gains also improve recovery of biologically meaningful patterns in a multifactorial DI-SPA screening data set compared with existing data processing workflows. We therefore reanalyzed the MitoTox data^5^ using ProjDIA, CsoDIAq, and CHIMERYS from the original DI-SPA study. CHIMERYS was also attempted as an external library-free workflow for this data set. The CHIMERYS log indicated that the raw files were processed and that preliminary search statistics were generated; however, the final global confidence-estimation step did not complete under the tested settings (Table S11). Therefore, CHIMERYS did not provide a final comparable *q* ≤ 0.01 PSM/protein table for the downstream MitoTox analysis. We interpret this outcome as a workflow-compatibility and output-availability limitation under the tested settings, not as evidence of inferior CHIMERYS performance. The MitoTox experiments studied toxin responses across genotypes (HEK 293T Wild-type or PPTC7 knockout (KO) cells) and nutrient backgrounds (DMEM and human plasma-like medium (HPLM)). In this setting, the key biological questions are not only how many proteins are detected, but also which proteins respond to toxin treatment overall and which proteins show genotype-dependent toxin responses. ProjDIA recovered 259 proteins with significant overall toxin effects, compared with 176 for CsoDIAq, and 49 proteins with significant genotype-specific toxin effects, compared with 18 for CsoDIAq (Figure 6a). These correspond to increases of 47.2% and 172.2%, respectively. These counts summarize the number of quantified proteins passing the specified statistical threshold, but they do not by themselves establish biological specificity. Accordingly, the biological interpretation is based on recovery of predefined toxin-associated response patterns rather than on total significant-protein counts alone.

**Figure 6:**
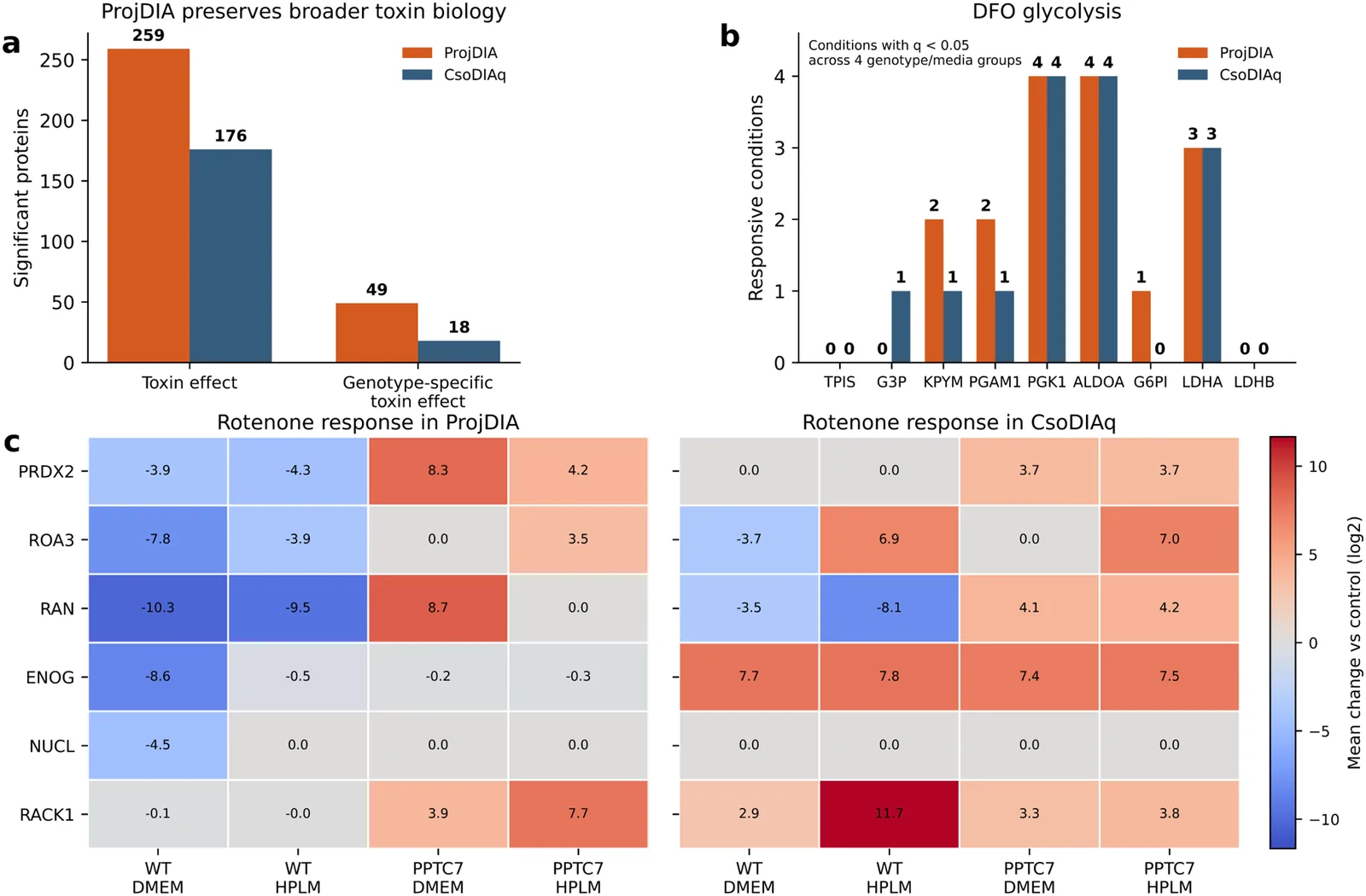
Recovery of predefined and exploratory toxin-response patterns in the MitoTox DI-SPA screen. (a) Descriptive screen-level counts of proteins with significant overall toxin effects or genotype-dependent toxin effects (*q* < 0.05). These counts summarize screen-level recovery and are not interpreted alone as evidence of biological validity. (b) Number of genotype/media backgrounds in which predefined DFO-associated glycolysis proteins showed significant DFO-versus-control differences (*q* < 0.05). Both workflows recovered the core DFO-associated glycolysis response, while ProjDIA recovered the response across more backgrounds for several markers. (c) Exploratory rotenone case study. Heatmaps show mean rotenone-versus-control log2 changes for six representative proteins across four genotype/media backgrounds, comparing ProjDIA and CsoDIAq. These proteins were selected post hoc and are shown as an illustrative response-pattern example rather than as a prespecified rotenone marker panel.

To anchor this comparison to a known biological signal, we next examined the toxin deferoxamine (DFO)-associated glycolysis response^35^ highlighted in the original DI-SPA study.^5^ For each glycolysis protein, we counted in how many of the four genotype/media backgrounds DFO produced a significant change relative to the matched control. In this analysis, a higher value indicates that the protein is recovered more consistently as a DFO-responsive marker across biological contexts. Both ProjDIA and CsoDIAq recovered the core glycolysis response, including recurrent DFO-associated changes in proteins such as PGK1, ALDOA and LDHA. However, both methods recovered KPYM and PGAM1 overall (Figure S8a); ProjDIA additionally resolved a significant response for G3P and G6PI, and recovered more responsive backgrounds for KPYM, PGAM1 and G6PI (Figure 6b). Thus, Figure 6b shows not the size of the DFO effect, but how consistently each method recovers known DFO-associated biology across the four experimental backgrounds. Detailed views of the DFO glycolysis marker set are shown in Figure S8. Figure S8a shows the mean DFO-versus-control response for individual glycolysis markers, confirming that both workflows recovered the core DFO-associated glycolysis pattern. When resolved by genotype and medium, these responses were clearly context dependent rather than uniform across all backgrounds, and ProjDIA preserved the expected glycolysis-associated pattern across more backgrounds for several markers (Figure S8b).

As a complementary toxin-specific case study, we next examined the influence of rotenone on protein with different genotypes. Condition-level heatmaps of six representative proteins showed that rotenone-versus-control responses were more clearly separated between WT and PPTC7 cells in the ProjDIA analysis than in CsoDIAq across the same genotype and medium backgrounds (Figure 6c). In particular, proteins such as PRDX2, ROA3, RAN, and RACK1 showed more distinct WT-versus-PPTC7 response patterns with ProjDIA, whereas the corresponding CsoDIAq responses were weaker or less consistently resolved across conditions. This case study provides a concrete example of how improved signal recovery by ProjDIA can translate into clearer preservation of biologically meaningful genotype-dependent toxin responses. Overall, these analyses show that the gains from ProjDIA extend beyond identification counts to improved recovery of multifactorial biological structure in DI-SPA screening data.

## Discussion

In DI-SPA, due to the lack of the separation step, many peptides are fragmented at the same time, leading to incredibly complex MS2 spectrum for each isolation window. In addition, small run-to-run mass shifts can make fragments matching less reliable. As a result, it presents significant challenge for reliable peptide mining. In this work, ProjDIA tackled this challenge via: (i) scores spectra in a way that also penalizes missing expected fragments, not only rewarding matched peaks; (ii) corrects mass error using a two-pass, high-confidence target set with robust estimates (median and MAD); (iii) reports monotone q-values, with a semisupervised rescoring step to improve separation of borderline matches while reducing the risk of overfitting. Across varied public DI-SPA data sets and acquisition settings, ProjDIA produced more confident identifications at the same q-value threshold than existing DI-SPA workflows. The ablation results show that the improvement does not come from one single innovation. Instead, each module helps most in the situation it was designed for: fragment-bin projection helps most when fragment peaks are less well resolved and more overlapped at lower MS2 resolution, while confidence-guided mass recalibration becomes more important as resolution increases, peak centroids become more specific, and small mass-error shifts become more limiting. Semisupervised rescoring provides a smaller but consistent additional gain, mainly by improving the ranking near the cutoff rather than changing the overall behavior of the search.

Because DI-SPA spectra are highly mixed, target–decoy behavior alone may not fully reflect false matches under strong interference. We therefore used species entrapment as external negative control. Under this test, ProjDIA’s q-values were generally well calibrated or slightly conservative, and entrapment remained low at commonly used thresholds (*q* ≤ 0.01). This matters in practical applications because DI-SPA is often carried out with many repeated measurements, where even small errors in inflation can accumulate. Consistent with this need, ProjDIA showed stable identification performance within large replicate series and stayed fast enough to handle large batches of data.

Our results suggest that the improved analytical performance of ProjDIA can support biological interpretation in rapid DI-SPA perturbation screens. In the MitoTox reanalysis, significant-protein counts were treated as descriptive screen-level summaries, while biological interpretation was anchored to predefined toxin-associated response patterns. The DFO glycolysis analysis provided the main anchored example, with ProjDIA recovering the expected response across more genotype and medium backgrounds for several markers. A complementary rotenone case study was retained as an exploratory example of genotype-dependent response-pattern recovery. Together, these findings suggest that improvements in scoring, recalibration, and confidence modeling can influence not only identification yield, but also the biological patterns that are recoverable from DI-SPA screening data sets.

ProjDIA also differs from previous DI-SPA workflows in its intended use case and algorithmic focus. Briefly, CsoDIAq is an established spectral-library workflow for DI-SPA analysis, whereas RE-FIGS improves repeated-acquisition DI-SPA by using repeated-feature behavior with machine-learning-based scoring and featured-ion-guided quantification. RE-FIGS was therefore not included as a primary end-to-end comparator across all benchmarks because its main advantage depends on repeated-acquisition-specific information and upstream CsoDIAq-style no-filter SSM output. In contrast, ProjDIA was designed to improve the core DI-SPA search and confidence model without requiring repeated-acquisition-specific information, using fragment-bin projection scoring, confidence-guided recalibration, monotone q-value reporting, and guarded semisupervised rescoring.

ProjDIA is a library-driven workflow. To improve usability, we designed it to accept spectral libraries in TSV/CSV table formats, MGF format and converted DIA-NN Parquet format, allowing users to work with both public libraries and custom resources. For DI-SPA, the disadvantages of needing a library are alleviated compared with the LC-MS case. As most of DI-SPA experiments focus on a defined set of targets, which reduces the need of an absolute ultradeep library. Compared with the library-free approaches, e.g., CHIMERYS, ProjDIA showed better protein coverage as well as better tolerance to the diverse quality of DI-SPA experiments.

Several limitations still need to be addressed in future iterations of the ProjDIA. First, performance at 30k and 45k MS2 resolution remains limited by reduced fragment resolution and peak overlap. ProjDIA improves matching robustness under these conditions but cannot remove the underlying loss of fragment specificity caused by lower resolving power. Second, ProjDIA is currently only demonstrated with Orbitrap instruments due to the majority of available DI-SPA data being collected with Orbitrap mass spectrometers. Therefore, performance of ProjDIA for experiments performed using other mass analyzers still needs to be validated and optimized as data becomes available. Third, while current ProjDIA can process LC-MS proteomics data in principle, its performance is not comparable with other state-of-the-art LC-MS DIA workflows. Given the significant potential of ProjDIA in handling complex DIA data, further efforts to expand the scope of ProjDIA to LC-MS experiments are warranted.

## Experimental Section

### Data and Software

#### Data Sets

We evaluated ProjDIA on public direct-infusion DIA (DI-SPA) benchmark data sets spanning common acquisition settings and replicate-heavy study designs. For acquisition-setting robustness, we analyzed a HeLa digest standard acquired across Orbitrap MS2 resolutions (30k–240k) and sample loads (0.125–1 μg), available from MassIVE under accession MSV000089546. To assess stability under repeated acquisition, we analyzed 100 consecutive DI-SPA acquisitions of the same MCF-7 sample acquired on an Orbitrap Lumos. To evaluate biological utility, we reanalyzed MitoTox DI-SPA data set from the original DI-SPA study, a multifactorial perturbation experiment spanning toxin treatment, genotype, and nutrient conditions, available from MassIVE under accession MSV000085156. To cover additional sources of data variation within a single controlled study, we analyzed data sets from the RE-FIGS study, including 12 repeated acquisitions of a HeLa digest standard, a defined three-species mixture (human, yeast, and *E. coli*). The corresponding raw data are available from ProteomeXchange^36^ under accession PXD036209, and the associated library files are available under accession PXD002952. For species-entrapment evaluation, we generated additional direct-infusion DIA data in this study. These raw files and associated library files have been deposited to ProteomeXchange under accession PXD076477. Raw files were converted to mzXML format using MSConvert^37^ with vendor peak picking enabled for MS1 and MS2, with default settings used for all other parameters.

#### Software, Confidence Threshold, and Benchmark Settings

ProjDIA searches centroided mzXML files against spectral libraries containing target entries with appended decoys and reports identifications using monotone q-values. Benchmark comparisons were performed against CsoDIAq 2.12 and CHIMERYS 4.0.0. For the library-based comparison, ProjDIA and CsoDIAq were run with the same spectral library to isolate workflow-level differences. CHIMERYS was included as a library-free comparator. Unless stated otherwise, identifications were reported at *q* ≤ 0.01 for each workflow output.

#### ProjDIA Settings

ProjDIA was run with ppm tolerance = 30, min shared peaks = 3, top-K query peaks = 0 (direct-infusion setting), max library peaks per precursor = 10, scan chunk size = 50, and score mode = MaCC with projected scoring enabled. Two-pass mass-error recalibration and semisupervised ML rescoring were enabled (ML iterations = 4), and protein inference (IDPicker) was enabled using peptides with *q* ≤ 0.01 for inference. The mass-error recalibration model was fitted using target PSMs with *q* ≤ 0.01, requiring ≥ 200 confident PSMs. Pass-2 matching used the recalibrated tolerance returned by the robust fit, capped so that it did not exceed the initial tolerance

#### CsoDIAq Settings

CsoDIAq 2.12 was run using recommended defaults, with peak-matching tolerance set to 30 ppm. Mass correction was enabled using the histogram-based correction option. Other settings were kept at defaults.

#### CHIMERYS Settings

CHIMERYS 4.0.0 was run in nondiscovery mode (Discovery Mode = FALSE) using the UniProt human-reviewed FASTA. Searches used Trypsin/P (full specificity), up to 2 missed cleavages, peptide length 7–30, precursor charge 1–4, fragment mass tolerance 20 ppm, variable modification M­[UNIMOD:35], and fixed modification C­[UNIMOD:4], using the inferys 4.7.0 fragmentation prediction model.

#### Library Format Support

ProjDIA accepts spectral libraries in TSV/CSV table formats­(OpenSWATH^38,39^ /SpectraST^40^ in TraML-style tables, including the Human-Pan public library^41,42^ and Fragpipe library generation output via EasyPQP^43^), MGF format from Skyline,^44^ and DIA-NN–derived Parquet libraries after conversion. For all data processing, we used a library file from the previous DI-SPA experiments.^22,23^

### Usability

ProjDIA includes a graphical user interface implemented in tkinter and is distributed as a standalone executable to support use without command-line interaction. A log window displays analysis progress and reports errors when they occur.

### Input Files, Window Grouping, Peak Pooling, Matching, and Projection Scoring

#### Input Files and Scan Grouping

ProjDIA reads centroided MS2 spectra in mzXML format using Pyteomics. For each spectrum, ProjDIA extracts the scan number, precursor (window-center) *m*/*z*, isolation-window width, and FAIMS compensation voltage (when present). MS2 scans are grouped by (windowCenterMz, windowWidth, compensationVoltage) to prevent pooling across different DIA windows and to avoid mixing FAIMS planes. For each scan, ProjDIA records the mzXML spectrum identifier (when available), precursor *m*/*z*, window width, retention time (when present), compensation voltage (when present), and total ion current (TIC).

#### Peak Preprocessing and Pooling

Within each window group, query peaks are pooled in scan chunks while tagging each peak with its originating scan number. In the direct-infusion benchmark setting, no top-*k* filtering was applied and peaks with nonpositive intensity were removed. Library fragments were capped per precursor by retaining the top max library fragments by intensity, and fragments from all precursors falling within the DIA window were pooled once per window. Pooled query and library peak lists were *m*/*z*-sorted prior to matching.

#### Fragment Matching and Candidate Formation

Candidate matches are formed by pairing pooled query peaks to pooled library peaks within a fragment mass tolerance using an *m*/*z*-ordered sweep. For each matched pair, ProjDIA stores the ppm mass error

1 $$\Delta_{\mathrm{ppm}}=\frac{(m/z{)}_{\mathrm{ref}}-{\left(m/z\right)}_{\mathrm{query}}}{(m/z{)}_{\mathrm{ref}}}\times 10^{6}$$

where (*m*/*z*)_ref_ is the library fragment *m*/*z* and (*m*/*z*)_query_ is the observed fragment *m*/*z*. Matched pairs are aggregated into candidate groups keyed by (libraryIdx’ scan). Candidates are required to meet a minimum shared-fragment threshold. This filter is applied after matching and reapplied after pass-2 mass-error filtering.

#### Projection Scoring and Ranking

For each candidate, ProjDIA projects observed query intensities into the library precursor’s fragment-bin layout to form a fixed-length query vector q aligned to the library vector l. Using square-root transformed intensities, the projected query entry for library fragment bin *j* is

2 $$q_{j}=\underset{i\in M\left(j\right)}{\max}\sqrt{I_{i}}$$

where M(j) is the set of matched query peaks assigned to bin *j*. If no query peak maps to bin *j*, then *q*_*j*_ = 0. This max-per-bin rule prevents multiple query peaks from inflating a single expected fragment position. Library entries use the corresponding transformed library intensities $l_{j}=\sqrt{I_{j}^{\mathrm{lib}}}$ . Similarity is computed as cosine similarity over the full library fragment layout:

3 $$\cos\left(l,q\right)=\frac{l\cdot q}{∥l∥∥q∥}$$

Candidates are ranked by a MaCC-style score:

4 $$\mathrm{MaCC}=\cos\left(l,q\right)\times {\left(\mathrm{shared}\right)}^{1/5}$$

which is used for downstream target-decoy confidence estimation and filtering unless ML rescoring is applied. Relative to matched-only similarity, this projected representation makes missing expected fragments contribute directly via *q*_*j*_ = 0 and limits score inflation through the max-per-bin rule.

### Target–Decoy Confidence Estimation and Monotone Q-Values

Candidates are ranked by a chosen score when MaCC is set by default, ML score when rescoring is enabled, and sorted from highest to lowest. Let *T*(*k*)­and *D*(*k*) denote the cumulative numbers of target and decoy matches among the top k-ranked entries.^45^ The running false discovery rate (FDR) is computed as

5 $$\mathrm{FDR}\left(k\right)=\frac{s\cdot D\left(k\right)}{\max\left(1,T\left(k\right)\right)}$$

where *s* is a decoy-scaling factor used only when the library target:decoy ratio deviates from 1:1 (*s* = *N*_*targets*_/*N*_*decoys*_).

Per-match q-values are reported as the minimum FDR at which the match would be accepted, computed by a reverse cumulative minimum:^46^

6 $$q\left(k\right)=\underset{j\geq k}{\min}\mathrm{FDR}\left(j\right)$$

Identifications were reported at *q* ≤ 0.01.

### Two-Pass Mass-Error Recalibration

#### Pass 1 (Initial Search)

Fragment matching and scoring are performed using the initial fragment tolerance (30 ppm in the benchmark setting), and q-values are computed from the pass-1 ranking.

#### Fit Set (Confidence Gate)

To estimate run-specific fragment mass error, ProjDIA fits a recalibration model using only target matches with *q* ≤ 0.01. Fitting is performed only when at least 200 confident target PSMs are available. For each candidate, ProjDIA summarizes fragment mass error by taking the median ppm error across its matched fragment pairs, producing one ppm value per candidate for fitting.

#### Robust Center and Spread

The ppm offset is estimated as the median of the fit-set ppm values,

7 $$\mu =\mathrm{median}\left(\Delta_{\mathrm{ppm}}\right)$$

and the spread is estimated using the median absolute deviation (MAD),^47^

8 $$\sigma \approx 1.4826\times \mathrm{median}\left(\left|\Delta_{\mathrm{ppm}}-\mu \right|\right)$$

If σ is nonfinite, a standard-deviation estimate is used as a fallback.

#### Pass-2 Tolerance and Rescore

The recalibrated tolerance is set to

9 $${\mathrm{tol}}_{2}=4\mathrm{ppm},3\sigma$$

and is capped not to exceed the initial tolerance. Matched fragment pairs are retained if |Δ_ppm_ – *μ*| ≤ tol_2_, candidates are rebuilt from the retained pairs, the minimum shared-fragment requirement is reapplied, and candidates are rescored. If pass 2 yields no candidates after filtering, results fall back to pass 1 for that run.

### Semisupervised ML Rescoring

ProjDIA optionally refines candidate rankings using a semisupervised ML rescoring based on logistic regression. Rescoring operates on the set of scored candidates after mass-error recalibration and uses a compact set of match-quality features computed during scoring: shared, cosineScore, maccScore, ppmAbsMean, ppmPenalty, explained, explainedAll, and ionCount.

Training labels are defined from the target-decoy framework. Decoys are used as negatives, and positives are selected from high-confidence targets (*q* ≤ 0.01) from the current iteration. Rescoring is performed for a small fixed number of iterations (4 by default). At each iteration, the model is retrained, and positives are reselected based on the updated ranking, which sharpens separation near the reporting threshold without changing the underlying match generation.

To limit in-sample optimism during semisupervised rescoring, ProjDIA does not use in-sample training scores as final reported scores. When rescoring is enabled, scan-grouped cross-validation is used: scan groups are assigned deterministically to folds, the rescoring model is trained on the remaining folds, and each scan group receives a held-out fold score from a model not trained on that scan group. Final q-values are then computed only after held-out fold scores have been assigned to all scored candidates. If the number of eligible target or decoy examples is below the rescoring guard, ML rescoring is skipped and the base score is retained for q-value calculation. For the entrapment holdout control, entrapment-origin entries remained searchable and reportable but were excluded from mass-error recalibration fitting and from semisupervised rescoring model fitting; entrapment-derived decoys were also excluded from rescoring model fitting.

### Protein Inference

Protein inference was performed after peptide-level q-value filtering (*q* ≤ 0.01) using an IDPicker-style parsimony approach. Accepted peptides were mapped to protein accessions using the library annotations, and ProjDIA reported the smallest set of proteins or protein groups that explained the accepted peptides. The default protein-group output includes groups supported by a single peptide. ProjDIA does not compute an independent protein-level FDR; therefore, reported protein groups should be interpreted as peptide-level-filtered protein groups rather than protein-FDR-controlled identifications. To evaluate protein-level error behavior, we performed a protein-level entrapment sensitivity analysis using different supporting-peptide requirements (Table S9).

### Label-Free Quantification

For multirun LFQ, ProjDIA exports peptide- and protein-level intensity tables after applying the reporting threshold (*q* ≤ 0.01) and removing decoys. Peptide intensity is taken from the fragment-evidence summary reported by ProjDIA (*ionCountSumPSM*). These values are arranged into a peptide-by-run matrix. Protein LFQ tables are then generated by aggregating peptide intensities using the inferred protein–peptide mapping, producing a simple summed protein table (“protein_sum”) and an optional MaxLFQ-style protein table (“protein_maxlfq”). MaxLFQ protein intensities are derived from the peptide matrix by working in log space and treating zeros as missing. Sample-to-sample offsets are estimated from pairwise median log-differences across peptides shared between samples, and protein quantities are obtained by solving for a set of sample intensities that best match those pairwise relationships. Final values are reported back in intensity space.

#### LFQ Accuracy in the Three-Species A/B Mixture

LFQ accuracy was evaluated using the exported peptide and protein matrices at *q* ≤ 0.01. Runs were assigned to conditions A or B based on run-name annotations. For summary statistics, intensities ≤ 0 were treated as missing. Within each condition, mean intensity, and CV (sample SD/mean; ddof = 1 when *n* > 1) were computed using observed replicates only. Features were evaluated only if quantified in ≥ 4 replicates in both A and B and if CV < 0.20 in both groups. Fold-change was computed as log2­(meanA/meanB). Expected log2­(A/B) ratios were defined by the mixture design (yeast: + 1, human: 0, *E. coli*: – 2), and accuracy was summarized as log2 ratio error (observed – expected), using median and MAD. For the matched CsoDIAq comparison in Table S10, CsoDIAq outputs were processed using the same raw files, spectral library, mass tolerance, MaxLFQ quantification, and downstream feature filters. Ratio errors were calculated using the same expected log2­(A/B) values and summary statistics as for ProjDIA.

#### HeLa Dilution-Series

Dilution-series runs were assigned to injected concentrations (0.125, 0.25, 0.5, 1.0 μg/μL) from run names. Zeros were treated as missing. For each feature, mean intensity was computed at each concentration, and monotonicity was assessed only for features quantified at all concentrations. Response was summarized by the slope of a linear fit (mean intensity vs concentration) and Spearman correlation (ρ). “Strongly monotonic” features were defined as slope >0 and ρ ≥ 0.8. For normalized response plots, each feature’s curve was scaled by its maximum across concentrations, and the median normalized curve was overlaid.

### Entrapment Evaluation

Entrapment peptides were labeled with a “TRAP-” prefix and decoys with a “DECOY-” prefix. Entrapment calculations excluded decoys. At a reporting threshold (t), accepted nondecoy identifications with (*q* ≤ *t*) were classified as target identifications (T) or entrapment identifications (E). The observed entrapment rate was calculated as

10 $$\left[\mathrm{Observed}\;\mathrm{entrapment}\;\mathrm{rate}=\frac{E}{T+E}\right]$$

Because this raw fraction does not account for unequal target and entrapment search-space sizes, we also calculated a database-size-adjusted entrapment-based false discovery proportion (FDP) estimate. The entrapment-to-target search-space ratio was defined as

11 $$\left[r=\frac{N_{\mathrm{entrapment}}}{N_{\mathrm{target}}}\right]$$

where (*N*_*target*_) and (*N*_*entrapment*_) are the numbers of searchable target and entrapment peptides, respectively, after removing shared target/entrapment peptide sequences when applicable. The entrapment-based FDP estimate was then calculated as

12 $$\left[\mathrm{Entrapment‐based}\;\mathrm{FDP}=\frac{E\left(1+\frac{1}{r}\right)}{T+E}\right]$$

PSM-level entrapment curves used all accepted nondecoy PSMs. Peptide-level entrapment curves collapsed identifications to unique peptide sequences by taking the minimum q-value per peptide after removing “TRAP-” and “DECOY-” prefixes. In this holdout control, entrapment-origin entries remained searchable and reportable but were excluded from mass-error recalibration fitting and from semisupervised rescoring model fitting. Entrapment-derived decoys were also excluded from rescoring model fitting. For CHIMERYS, entrapment membership was assigned after searching by mapping accepted peptide sequences to in-silico tryptic digests of the target and entrapment proteomes, with shared target/entrapment sequences excluded. This CHIMERYS analysis was interpreted as a workflow-level postsearch entrapment diagnostic rather than an audit of the proprietary internal FDR procedure.

### Biological Reanalysis of the MitoTox Screen

We reanalyzed the public MitoTox direct-infusion DIA data set from the original DI-SPA study using ProjDIA and CsoDIAq. Sample annotations were taken from the accompanying metadata, and each sample was assigned genotype, medium, treatment, time point and replicate. CHIMERYS was also attempted for this data set; however, the final global confidence-estimation step did not complete under the tested settings, and no final comparable *q* ≤ 0.01 PSM/protein table was available for the downstream protein-level analysis. This outcome was recorded as a workflow audit rather than interpreted as a performance comparison. For each run, protein-level profiles were generated from the workflow outputs after identification filtering. For ProjDIA, we used the protein table inferred from peptides passing *q* ≤ 0.01 and extracted ionCountSum; for CsoDIAq, we used the proteinFDR output after removing decoys and duplicate scan–peptide–protein entries. Protein groups containing multiple human UniProt entries were split equally across matched proteins. Within each run, protein signals were normalized by the total assigned signal and transformed into log2-relative abundance values.

To quantify preservation of the multifactorial biological structure, we fitted protein-wise linear models including treatment, genotype, and medium terms, together with treatment × genotype and treatment × medium interactions. Significance for each effect class was assessed by comparing full and reduced models, and P values were adjusted using the Benjamini–Hochberg procedure; proteins with *q* < 0.05 were counted as significant. For the DFO glycolysis analysis, we tested DFO-versus-control differences within each genotype–medium background and counted, for each marker, in how many of the four backgrounds a significant response was recovered (*q* < 0.05). For the representative rotenone case study, 6 h treatment-versus-control responses were summarized as mean log2 changes within each genotype–medium background and visualized as heatmaps for six representative proteins. All downstream biological analyses and plotting were performed in Python.

### Runtime and Computer Environment

Runtime was measured as wall-clock time for end-to-end processing, excluding file download. All analyses were run on Windows 11 using Pro 64-bit with a processor Intel Core i7–14700 (28 cores), 32 GB RAM, and no GPU. CHIMERYS was evaluated through the cloud-based MSAID Platform trial; the CHIMERYS time was taken from the logged analysis-stage runtime after data submission and is reported only as cloud-platform operational context because the underlying hardware, cloud resource allocation, and account-level settings were not controlled by the authors.

### Direct Infusion Mass Spectrometry

HeLa standard digest was prepared in water containing 0.1% formic acid. The digested peptides were analyzed on an Orbitrap Exploris 480 using a direct-infusion method operated in data-independent acquisition (DIA) mode. Samples were ionized via cVSSI^48–50^ or ESI as the ion source, and ions were introduced into the mass analyzer through a heated ion transfer capillary maintained at 320 °C. DIA was performed in positive-ion mode using an MS2 resolution setting of 120,000 to ensure high mass accuracy. The AGC target was set to 300%, and the maximum injection time was set to Auto to optimize ion accumulation while maintaining an efficient acquisition duty cycle. DIA was acquired using a narrow isolation window of 1 *m*/*z* across the 380–1000 *m*/*z* range. The total acquisition time was approximately 3 min. Raw files were then converted to mzXML and analyzed for entrapment evaluation.

## Supplementary Material



## Data Availability

Previously published DI-SPA raw data and associated library files reanalyzed in this study are publicly available from MassIVE under accessions MSV000089546 and MSV000085156, and from ProteomeXchange under accessions PXD036209 and PXD002952. Additional direct-infusion DIA raw data and associated three-species spectral library files (yeast, human and *E. coli*) generated in this study have been deposited to ProteomeXchange under accession PXD076477. ProjDIA source code is available at GitHub (https://github.com/herbiyuliantoro/ProjDIA) and archived on Zenodo at 10.5281/zenodo.19373367. Scripts used to generate the figures and tables are included in the archived release. For reproducibility and peer review, ProjDIA is distributed as both a compiled standalone application and source code corresponding to the archived release. The Zenodo archive includes version information, documentation and a minimal reproducibility example with input files and expected outputs. The exact version used in this manuscript is ProjDIA v1.0, released under the MIT license.

## Notes

Additional module-ablation plots, retention heatmaps, peptide-overlap analysis, fragment-level evidence diagnostics, repeated-acquisition robustness analysis, machine-learning score and q-value diagnostics, three-species LFQ ratio–abundance analysis, DFO glycolysis recovery analysis, workflow-comparison table, data set-source table, PSM-to-peptide collapse metrics, paired replicate-level statistics, fragment-diagnostic tables, entrapment FDP calculations, recalibration/rescoring safeguards, protein-level entrapment sensitivity analysis, LFQ comparison table, and CHIMERYS/MitoTox output audit (PDF)
