# Supplementary material for: ProjDIA: Fragment-Bin Projection Scoring and Calibrated Confidence for Direct Infusion DIA Proteomics
Source: ACS Meas Sci Au. 2026 Jul 30;6(5):1571–85. doi: 10.1021/acsmeasuresciau.6c00119 (PMC13750382; doi:10.1021/acsmeasuresciau.6c00119)
Supplement: Supplementary file 1 [file tg6c00119_si_001.pdf]

Supplementary Figures

# ProjDIA: Fragment-Bin Projection Scoring and Calibrated Confidence for Direct Infusion DIA Proteomics

Herbi Yuliantoro[1], Peng Li[1]*

[1] Department of Chemistry, Lehigh University, 6 East Packer Avenue, Bethlehem, Pennsylvania 18015, United States

* Correspondence and requests for materials should be addressed to P.L. (pel624@lehigh.edu)

Contents

Figure S1. Identification yield across MS2 resolution under module ablations.

Figure S2. Retention of full-pipeline identifications after disabling individual modules.

Figure S3. Fragment-level evidence supporting ProjDIA and CsoDIAq peptide assignments.

Figure S4. Peptide-level overlap among ProjDIA, CsoDIAq, and CHIMERYS for representative matched benchmark subsets.

Figure S5. Robustness under 12 repeated DI-SPA acquisitions in the HeLa dataset.

Figure S6. Machine-learning score separation and q-value filtering assessed by decoy and species entrapment.

Figure S7. Species-specific LFQ ratio–abundance patterns for the three-species mixture.

Figure S8. Condition-level recovery of DFO-associated glycolysis responses across genotype and nutrient backgrounds.

Table S1. Summary of CsoDIAq, RE-FIGS, and ProjDIA for DI-SPA analysis.

Table S2. Source datasets used for each ProjDIA analysis.

Table S3. PSM-to-peptide collapse metrics for the Figure 2 benchmark datasets.

Table S4. Paired replicate-level statistics for Figure 2 benchmark comparisons.

Table S5. Fragment-level diagnostic summary for representative benchmark subsets.

Table S6. ProjDIA fragment-sharing multiplicity within isolation windows.

Table S7. Species-entrapment search-space sizes, accepted peptide counts, observed entrapment rates, and entrapment-based FDP estimates.

Table S8. Recalibration and rescoring safeguards against double-dipping.

Table S9. Protein-level entrapment sensitivity in the ten-run species-entrapment analysis.

Table S10. Comparison of ProjDIA and CsoDIAq LFQ ratio accuracy for the three-species mixture under matched post-processing filters.

Table S11. CHIMERYS output audit for the MitoTox dataset.

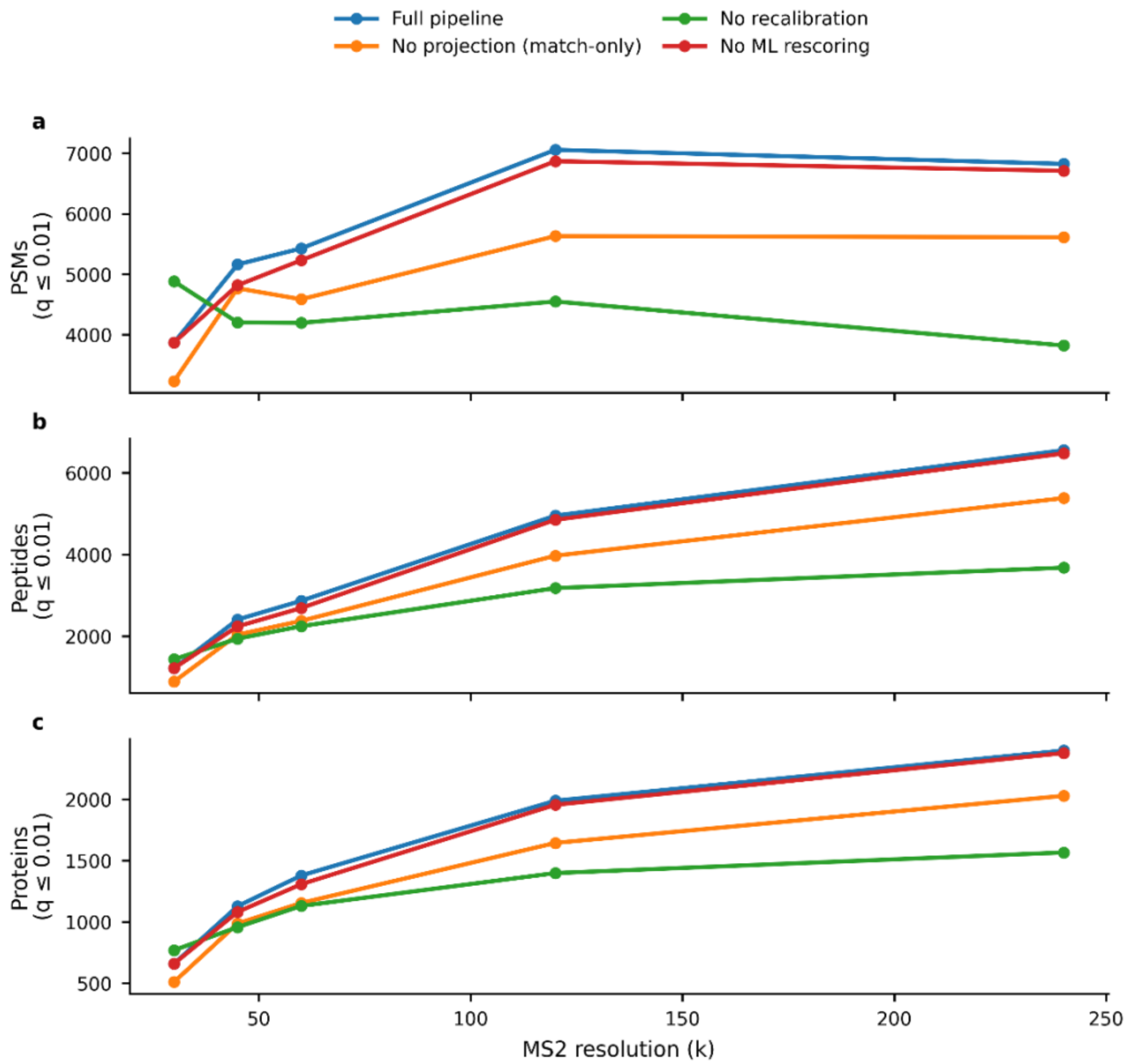


**Figure S1 | Identification yield across MS2 resolution under module ablations.** Numbers of confident identifications (q ≤ 0.01) reported by the full ProjDIA pipeline and by runs with individual modules disabled among MS2 resolution settings. (a) PSMs, (b) peptides, and (c) proteins. Ablations remove fragment-bin projection scoring ("No projection"), two-pass mass-error recalibration ("No recalibration"), or machine-learning rescoring ("No ML rescoring"). Within all levels, the full pipeline yields the most identifications, while each ablation reduces yield. The impact of disabling mass-error recalibration increases at higher resolution, whereas projection scoring contributes consistently, ML rescoring provides additional gains, most evident near the decision boundary.

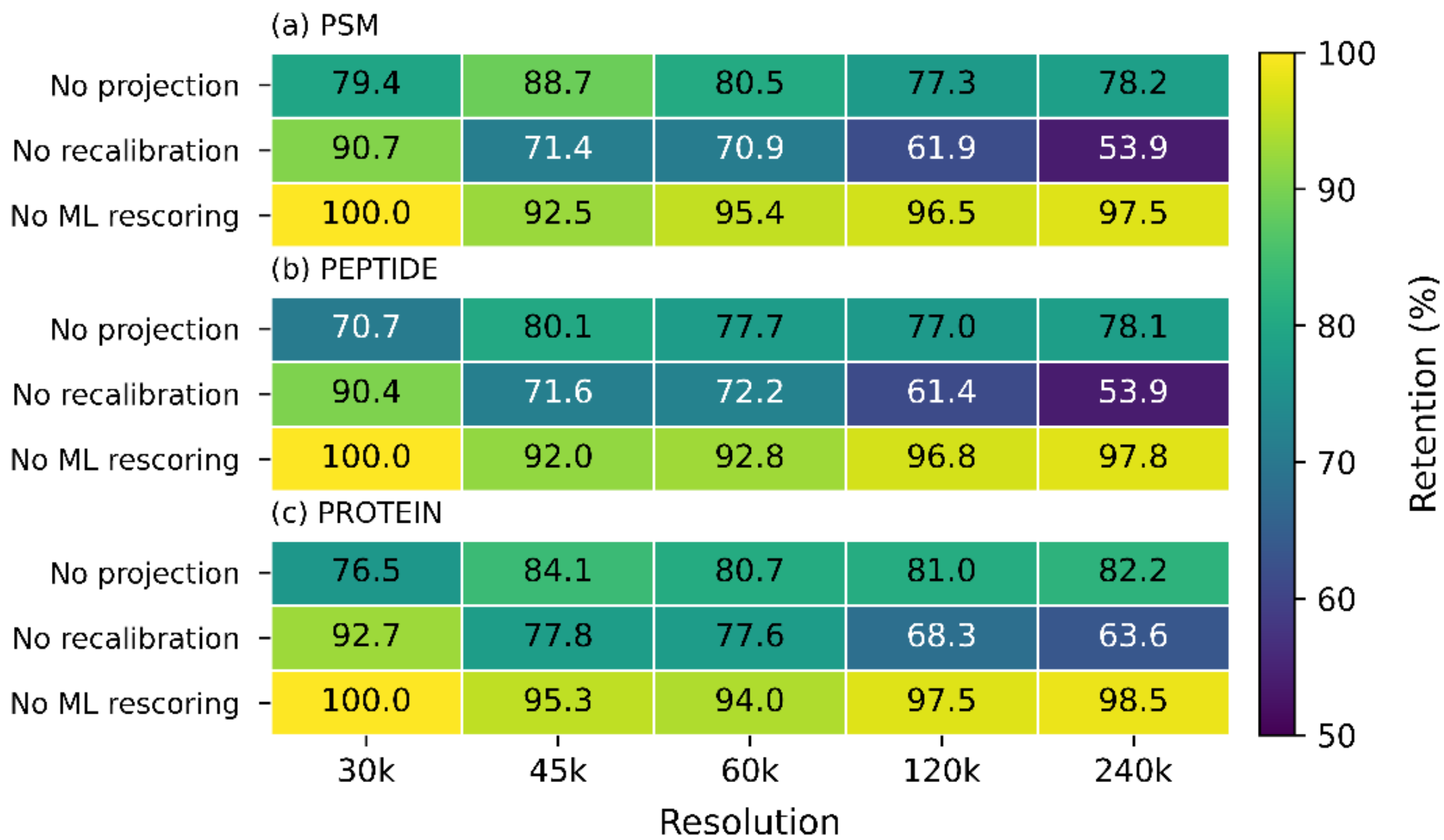


**Figure S2 | Retention of full-pipeline identifications after disabling individual modules.** Heatmaps show retention (%) of identifications when a single module is removed, computed as (shared with Full) / (Full total) at $q \leq 0.01$ for (a) PSMs, (b) peptides, and (c) proteins across MS2 resolutions (30k-240k). Removal of ML rescoring retains most full-pipeline IDs, indicating rescoring primarily refines borderline calls, whereas removal of mass-error recalibration results in the largest losses at higher resolution. Projection scoring removal causes a moderate but consistent reduction over resolutions, supporting a complementary contribution of scoring, recalibration, and rescoring to the final confident set.

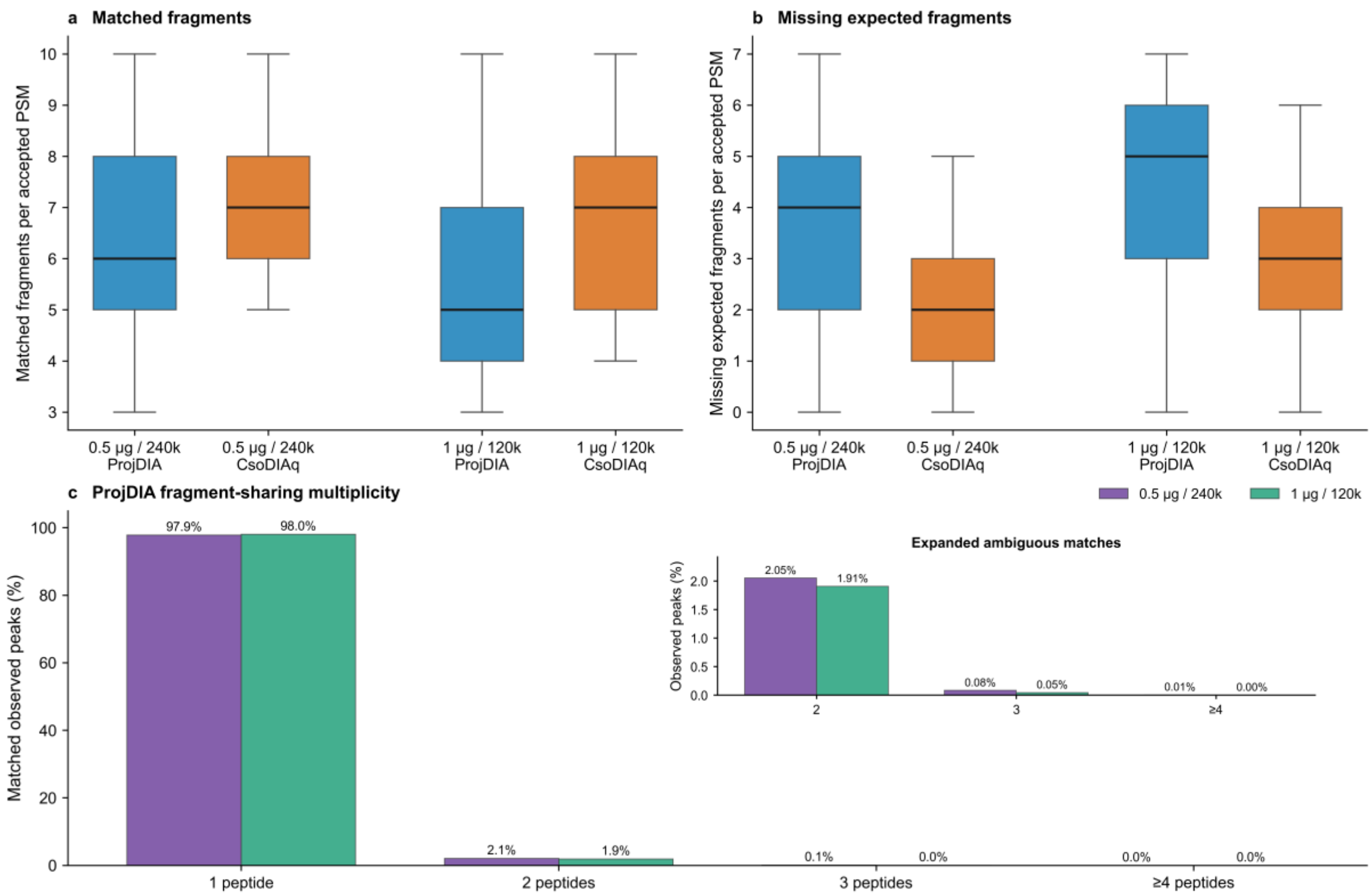


**Figure S3 | Fragment-level evidence supporting ProjDIA and CsoDIAq peptide assignments**. Fragment-level metrics were evaluated for accepted target PSMs at $q \leq 0.01$ using two representative benchmark subsets: 0.5 µg/240k and 1 µg/120k. (a) Matched fragments per accepted PSM. This panel shows how many experimental fragment peaks supported each accepted peptide assignment in ProjDIA and CsoDIAq. (b) Missing expected fragments per accepted PSM. This panel shows how many expected library fragments were not observed for each accepted assignment. Missing fragments were calculated as expected library fragments minus matched fragments. For CsoDIAq, the expected-fragment count was taken from the Peaks(Library) field. For ProjDIA, the expected-fragment count was approximated using the benchmark setting of up to 10 retained library fragments per precursor because this value was not reported as a separate column in the PSM-level output. (c) ProjDIA fragment-sharing analysis. This panel shows how often the same observed fragment peak was compatible with one or more accepted peptide candidates in the same isolation window. Most observed peaks were compatible with only one accepted peptide candidate, whereas peaks shared by two or more candidates were rare. The inset expands the low-abundance shared categories. CHIMERYS was not included in this fragment-level diagnostic because equivalent expected-library-fragment information was not available from the library-free.

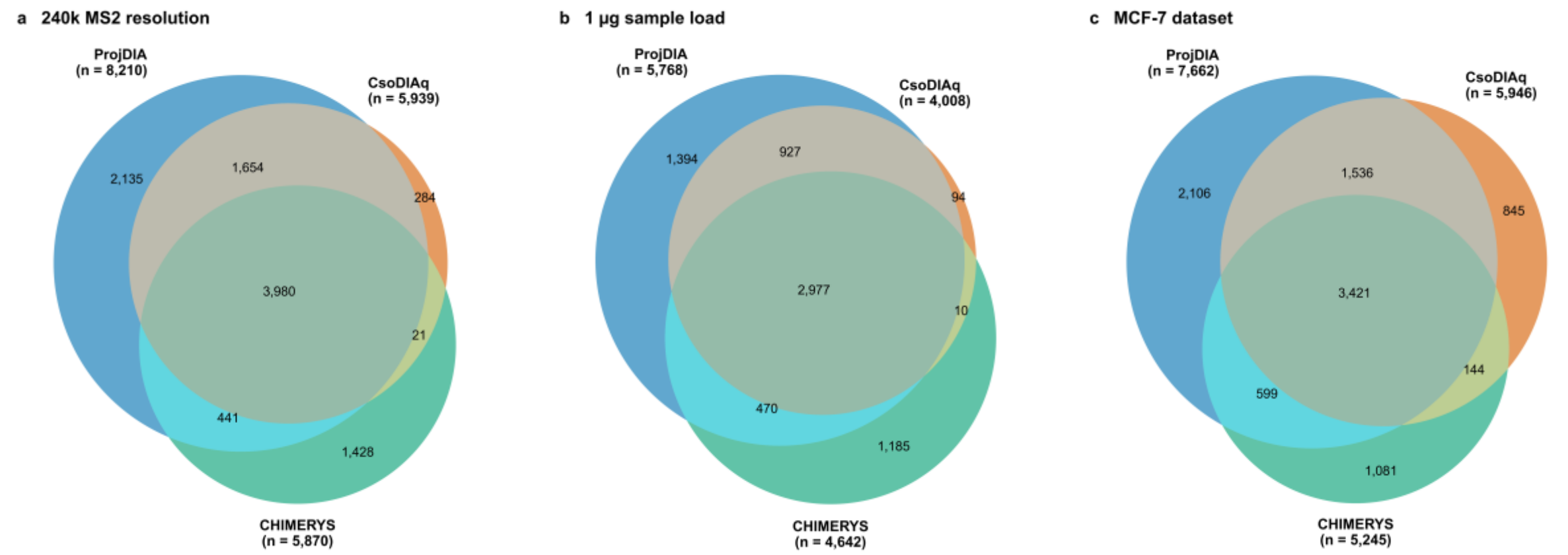


**Figure S4 | Peptide-level overlap among ProjDIA, CsoDIAq, and CHIMERYS for representative matched benchmark subsets**. Venn diagrams show overlap of unique peptide sequences reported at $q \leq 0.01$ across the three workflows for (a) the 0.5 µg, 240k MS2-resolution subset, (b) the 1 µg, 120k sample-load subset, and (c) a representative 20-run subset from the 100-run MCF-7 dataset. For the MCF-7 panel, peptide lists from the matched 20 runs were pooled within each workflow and deduplicated before calculating between-workflow overlap. Peptide sequences were normalized before comparison by removing modification annotations, charge suffixes, retaining amino acid letters only. Overlap was assessed at the peptide-sequence level rather than as direct scan-by-scan reassignment because the workflows differ in search paradigm, scoring strategy, and PSM reporting, particularly for the library-free CHIMERYS workflow.

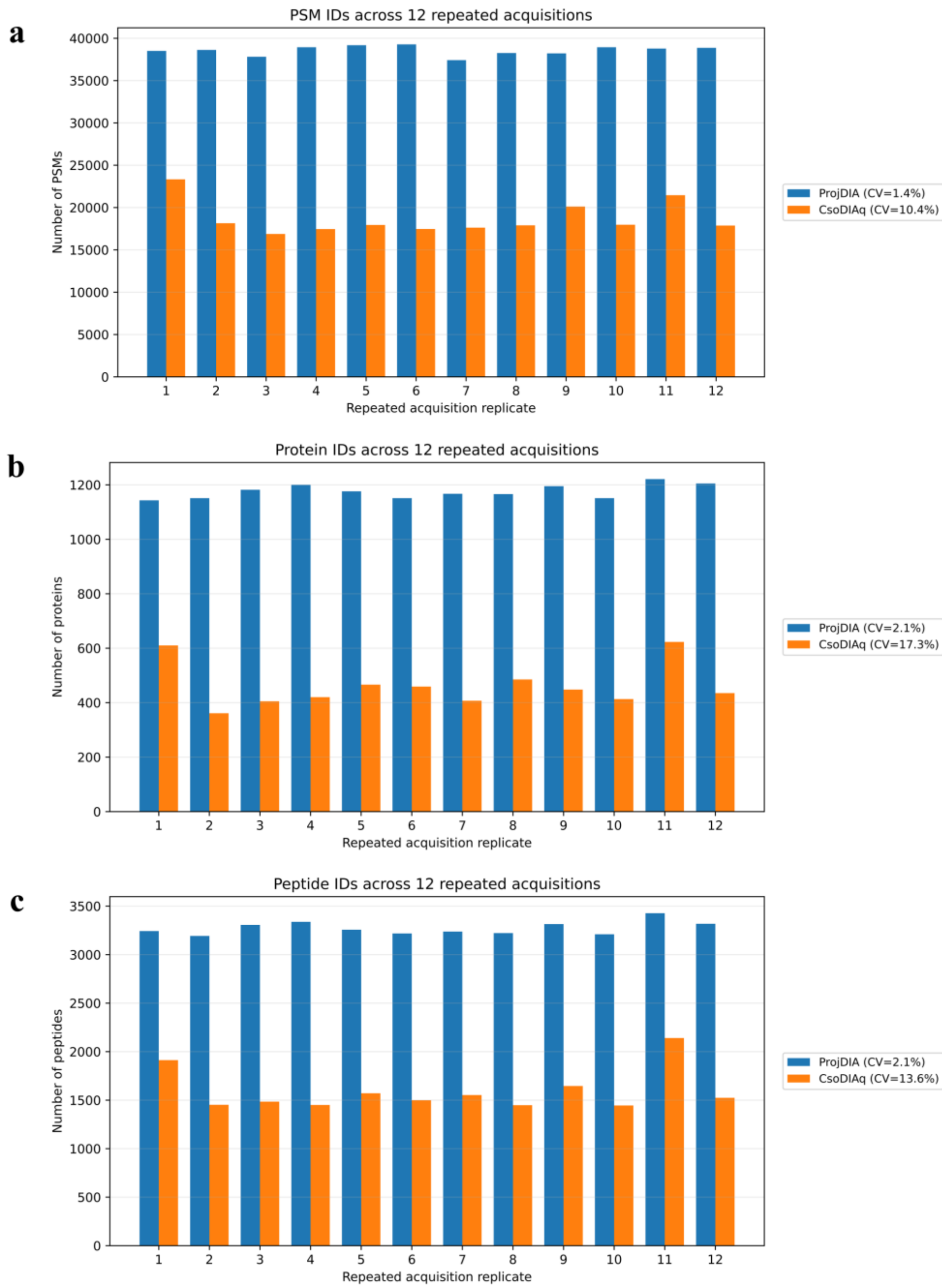


**Figure S5 | Robustness under 12 repeated DI-SPA acquisitions in the HeLa dataset.** Numbers of confident identifications ($q \leq 0.01$) for (a) PSMs, (b) peptides and (c) proteins from 12 repeated acquisitions of a HeLa digest standard from the RE-FIGS study, comparing ProjDIA and CsoDIAq. Bars show per-run counts.

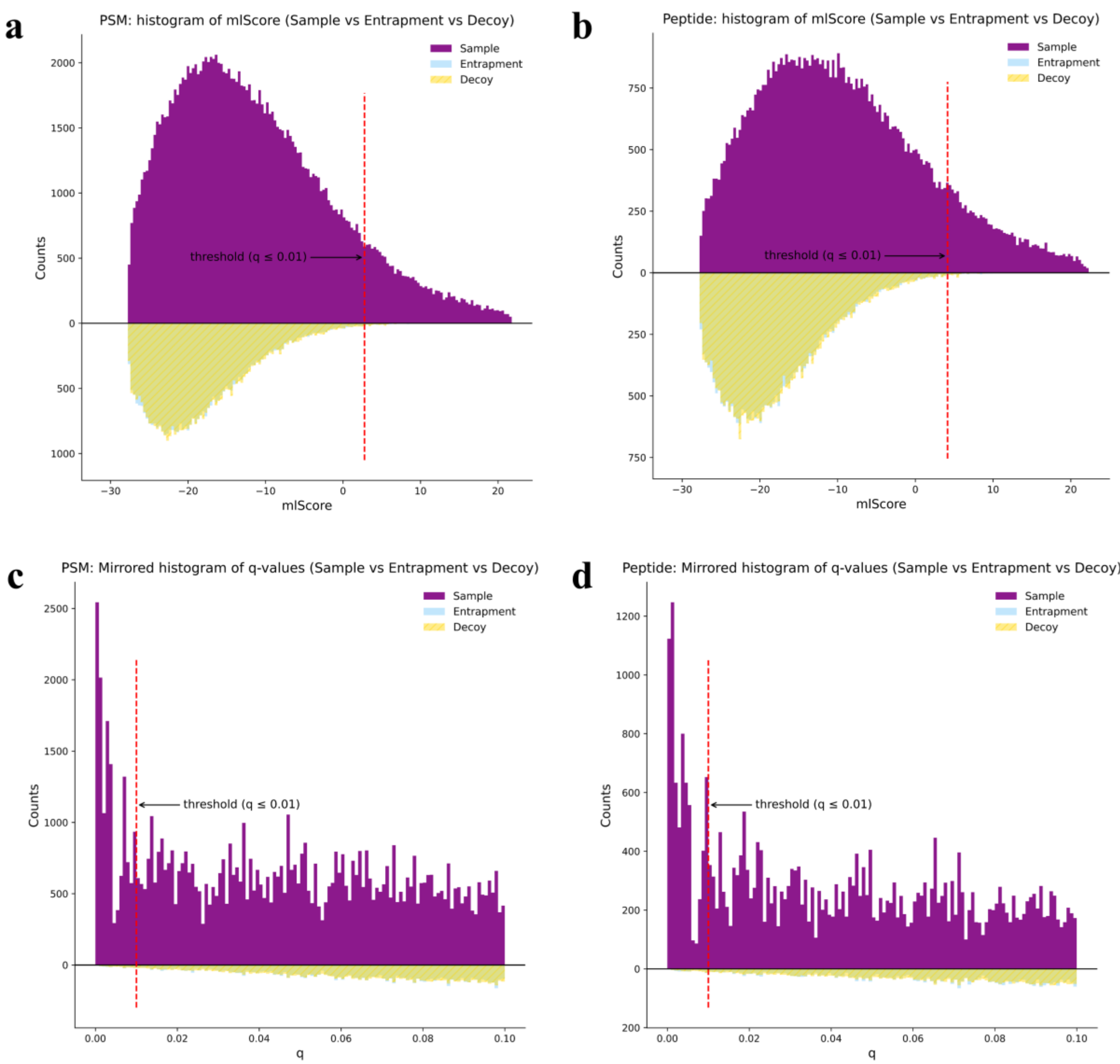


**Figure S6 | Machine-learning score separation and q-value filtering assessed by decoy and species entrapment.** Mirrored histograms summarize discrimination and confidence calibration using a composite library containing sample targets, species-entrapment entries (TRAP, absent-species peptides), and decoys. (a,b) Distributions of the ML-rescored score (mIScore) for PSMs (a) and peptides (b): sample targets are plotted above the axis, while entrapment and decoy matches are mirrored below. The red dashed line indicates the mIScore boundary corresponding to the $q \leq 0.01$ reporting set (minimum mIScore among non-decoy identifications with $q \leq 0.01$). (c,d) Corresponding mirrored histograms of q-values for PSMs (c) and peptides (d). The red dashed line marks the standard reporting threshold ($q = 0.01$). Depletion of entrapment and decoy matches below $q \leq 0.01$ supports effective discrimination and error filtering after rescoring.

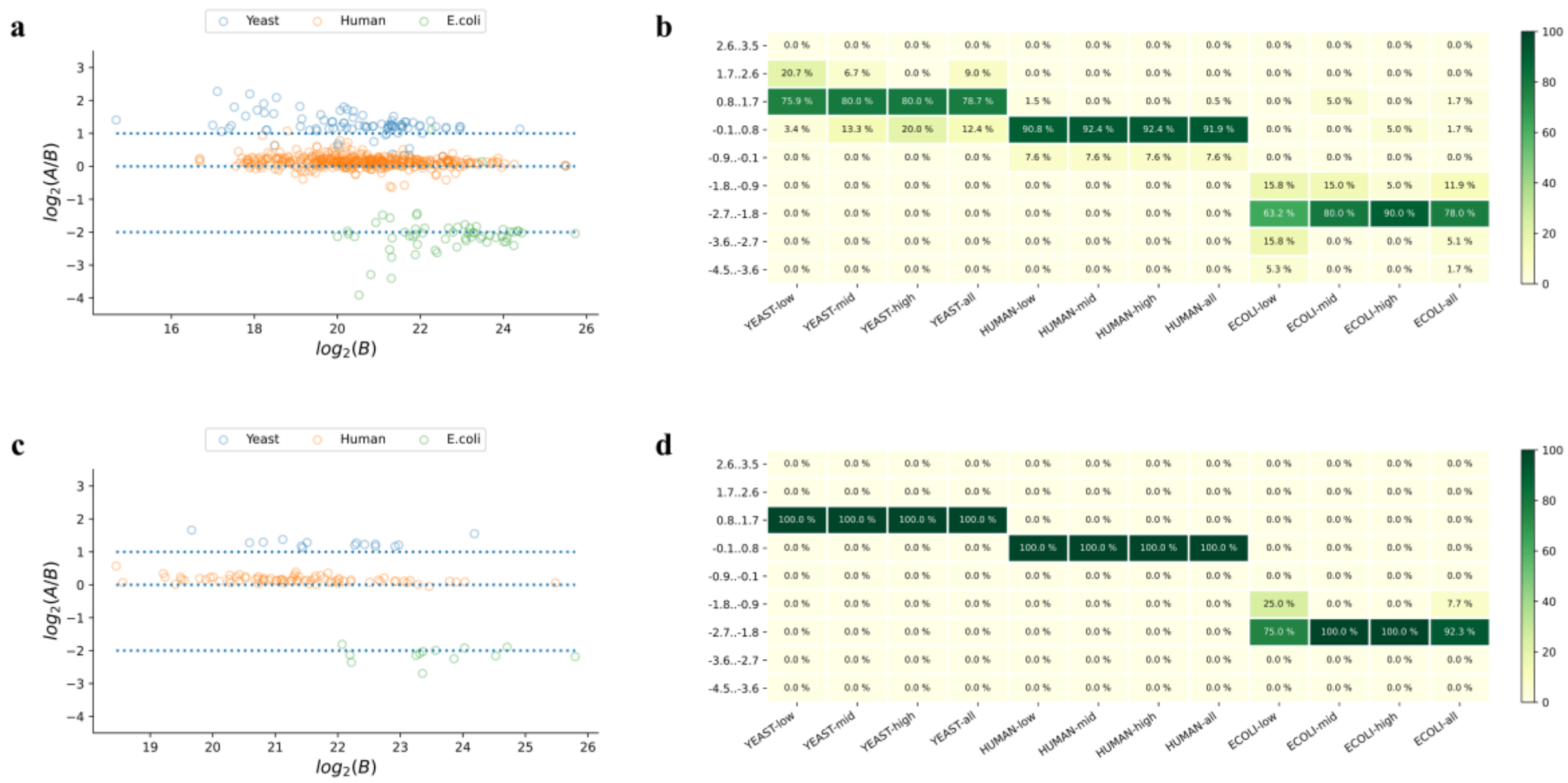


**Figure S7 | Species-specific LFQ ratios abundance for the three-species mixture.** Scatter plots show log2(A/B) versus log2(B) for quantified features passing the LFQ stability filters (≥4 replicates per condition and CV < 0.20 in both A and B), colored by species (yeast, human, E. Coli). Dotted horizontal lines mark the expected log2 fold-changes from the mixing design (yeast = +1, human = 0, E. Coli = −2). Heatmaps summarize the percentage of features falling into log2(A/B) bins (rows) across log2(B) abundance bins (columns), providing an abundance-stratified view of ratio separation by species. Panels show peptide-level quantification (a,b) and protein-level quantification (c,d).

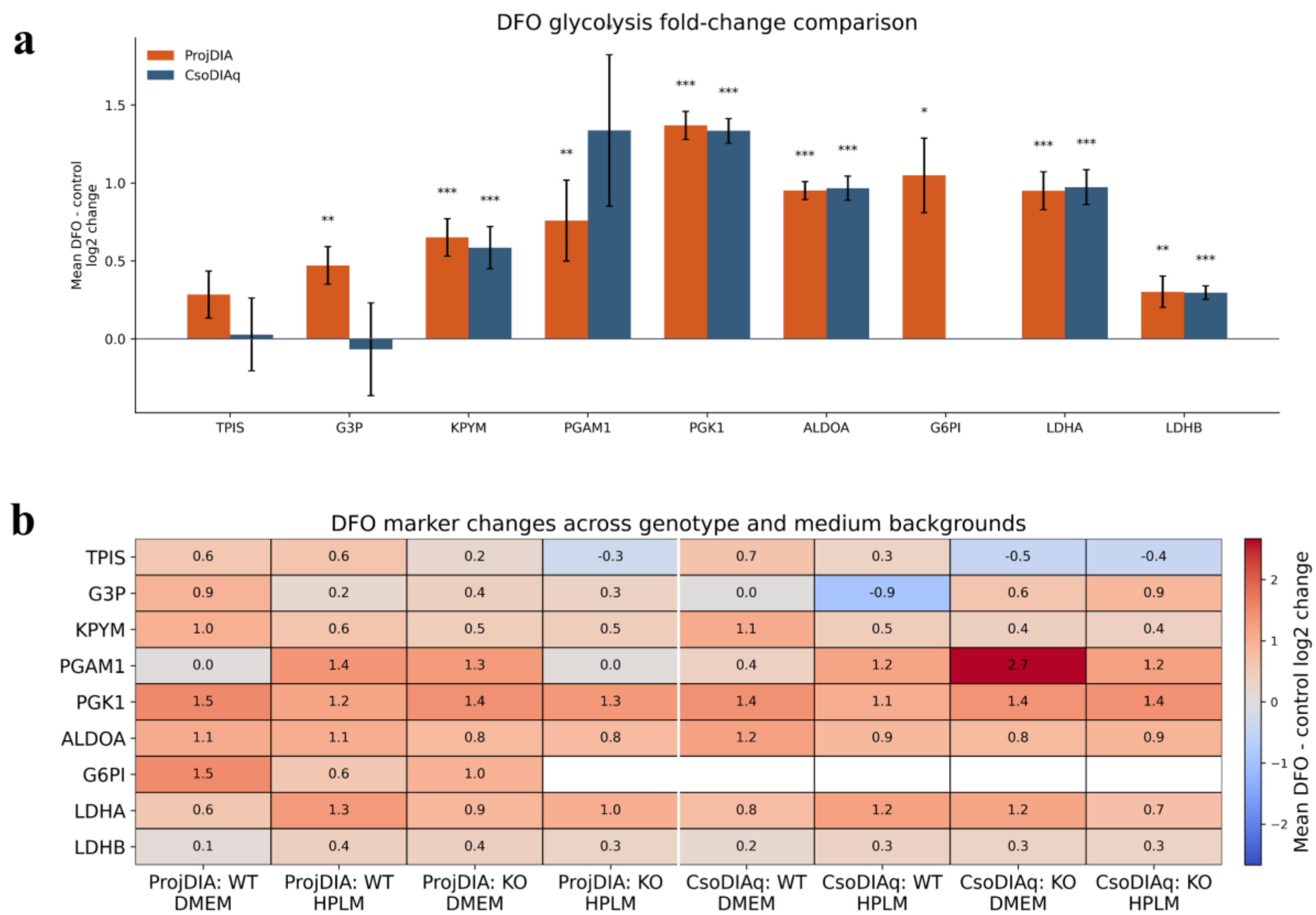


**Figure S8 | Condition-level recovery of DFO-associated glycolysis responses across genotype and nutrient backgrounds.** (a) Mean DFO-versus-control log2 fold-change for glycolysis marker proteins recovered by ProjDIA and CsoDIAq. Bars show the average response for each marker across the relevant biological backgrounds, and error bars indicate variability across backgrounds. Asterisks indicate significance for the DFO-versus-control comparison after multiple-testing correction: * $q < 0.05$, ** $q < 0.01$, *** $q < 0.001$ (b) Background-specific DFO-versus-control log2 fold-changes for the same glycolysis marker panel across the four genotype/media conditions (WT-DMEM, WT-HPLM, PPTC7-KO-DMEM and PPTC7-KO-HPLM) for each analysis workflow. This finding shows that the DFO effect is not uniform across all biological contexts, but ProjDIA generally preserves the expected glycolysis-associated response across a broader range of backgrounds, whereas CsoDIAq shows weaker, absent, or directionally mixed responses for several markers in specific conditions. It supports that ProjDIA recovers the DFO glycolysis biology more completely and provides a richer biological readout in this DI-SPA reanalysis.

**Table S1. Summary of CsoDIAq, RE-FIGS, and ProjDIA for DI-SPA analysis.**

| Feature | CsoDIAq | RE-FIGS | ProjDIA |
|---|---|---|---|
| Main role | General DI-SPA spectral-library search | Repeated-acquisition enhancement for DI-SPA | General DI-SPA search and confidence-calibration workflow |
| Repeated acquisition | Not required | Required/central to the strategy | Not required |
| Main strategy | Matched-fragment MaCC-style scoring | Repeated-feature behavior with ML-based scoring | Fragment-bin projection scoring with missing-fragment penalty |
| Practical trade-off | Established DI-SPA baseline | Can improve repeated-acquisition data, but requires repeated acquisition and upstream CsoDIAq-style no-filter SSM output | Preserves rapid DI-SPA use while improving scoring/confidence |
| User accessibility | Open-source DI-SPA software installation but requires software-environment setup | Script-based workflow requiring command-line use and takes more time for analysis | Standalone executable with graphical interface |

**Table S2. Source datasets used for each ProjDIA analysis.**

| Analysis section | Figure(s) | Dataset | Data source |
|---|---|---|---|
| Module-ablation analysis | Fig. S1, Fig. S2 | HeLa digest DI-SPA data acquired across MS2 resolutions | MassIVE MSV000089546 |
| Peptide identification across MS2 resolution and sample load | Fig. 2, Fig. S3; Fig. S4 | HeLa digest standard acquired across Orbitrap MS2 resolutions and sample loads | MassIVE MSV000089546 |
| 100-run replicate stability and runtime analysis | Fig. 3; Fig. S4 | 100 consecutive DI-SPA acquisitions of an MCF-7 sample | MassIVE MSV000085156 |
| Repeated-acquisition robustness | Fig. S5 | 12 repeated DI-SPA acquisitions of a HeLa digest standard from the RE-FIGS study | ProteomeXchange PXD036209 |
| Species-entrapment evaluation | Fig. 4A, Fig. S6 | Newly generated HeLa direct-infusion DIA data from our lab | ProteomeXchange PXD076477 |
| Species-entrapment evaluation (HeLa resolution series) | Fig. 4B,C | HeLa digest standard acquired across Orbitrap MS2 resolutions | MassIVE MSV000089546 |
| Three-species LFQ mixture analysis | Fig. 5A,B; Fig. S7 | Human, yeast, and E. coli defined three-species mixture from the RE-FIGS study | ProteomeXchange PXD036209; associated library PXD002952 |
| HeLa dilution-series LFQ analysis | Fig. 5C–F | HeLa dilution series from the RE-FIGS study | ProteomeXchange PXD036209; associated library PXD002952 |
| MitoTox biological reanalysis | Fig. 6, Fig. S8 | Multifactorial MitoTox DI-SPA perturbation dataset | MassIVE MSV000085156 |

**Table S3. PSM-to-peptide collapse metrics for the benchmark datasets.**

| Analysis | Condition | Tool | Mean PSMs | Mean unique peptides | PSMs per unique peptide | Unique peptides per 1000 PSMs |
|---|---|---|---|---|---|---|
| MS2 resolution | 30k | ProjDIA | 3978.5 | 1311.0 | 3.04 | 329.01 |
| MS2 resolution | 30k | CHIMERYS | 5874.0 | 1575.5 | 3.73 | 268.41 |
| MS2 resolution | 30k | CsoDIAq | 4020.0 | 1205.0 | 3.34 | 299.55 |
| MS2 resolution | 45k | ProjDIA | 5191.0 | 2376.5 | 2.18 | 457.95 |
| MS2 resolution | 45k | CHIMERYS | 6112.5 | 2321.5 | 2.63 | 379.81 |
| MS2 resolution | 45k | CsoDIAq | 4423.0 | 1957.0 | 2.26 | 443.18 |
| MS2 resolution | 60k | ProjDIA | 5847.5 | 2682.5 | 2.20 | 463.97 |
| MS2 resolution | 60k | CHIMERYS | 5984.5 | 2474.0 | 2.44 | 416.69 |
| MS2 resolution | 60k | CsoDIAq | 4682.0 | 2224.0 | 2.12 | 480.59 |
| MS2 resolution | 120k | ProjDIA | 6740.0 | 4792.3 | 1.41 | 711.22 |
| MS2 resolution | 120k | CHIMERYS | 6545.0 | 3939.3 | 1.66 | 601.91 |
| MS2 resolution | 120k | CsoDIAq | 5192.7 | 3538.7 | 1.47 | 681.29 |
| MS2 resolution | 240k | ProjDIA | 6907.7 | 6640.7 | 1.04 | 961.33 |

| | | | | | | |
|---|---|---|---|---|---|---|
| MS2 resolution | 240k | CHIMERYS | 6359.7 | 5056.7 | 1.26 | 795.14 |
| MS2 resolution | 240k | CsoDIAq | 4907.3 | 4687.3 | 1.05 | 955.50 |
| Sample load | 0.125 µg | ProjDIA | 5180.5 | 3727.5 | 1.39 | 719.44 |
| Sample load | 0.125 µg | CHIMERYS | 4926.5 | 3086.5 | 1.60 | 626.50 |
| Sample load | 0.125 µg | CsoDIAq | 3929.5 | 2784.5 | 1.41 | 708.61 |
| Sample load | 0.25 µg | ProjDIA | 6949.0 | 4899.0 | 1.42 | 705.00 |
| Sample load | 0.25 µg | CHIMERYS | 6535.5 | 4021.5 | 1.63 | 615.31 |
| Sample load | 0.25 µg | CsoDIAq | 5177.0 | 3519.5 | 1.47 | 679.89 |
| Sample load | 0.5 µg | ProjDIA | 6615.5 | 4736.5 | 1.40 | 716.00 |
| Sample load | 0.5 µg | CHIMERYS | 6585.5 | 4016.5 | 1.64 | 609.89 |
| Sample load | 0.5 µg | CsoDIAq | 5206.5 | 3496.0 | 1.49 | 671.11 |
| Sample load | 1 µg | ProjDIA | 7034.0 | 5047.0 | 1.39 | 717.45 |
| Sample load | 1 µg | CHIMERYS | 6610.0 | 4130.0 | 1.60 | 624.92 |
| Sample load | 1 µg | CsoDIAq | 5048.0 | 3500.0 | 1.44 | 693.33 |

Values are means across replicate acquisitions. These metrics clarify that PSM and unique-peptide counts do not necessarily scale linearly because multiple spectrum-level matches can collapse to the same peptide sequence.

**Table S4. Paired replicate-level statistics for Figure 2 benchmark comparisons**.

(A) HeLa sample-load benchmark at 120K MS2 resolution. (B) HeLa MS2-resolution benchmark at 0.5 µg sample load. Differences were calculated as ProjDIA minus the comparator workflow within matched public raw acquisitions processed by each workflow. Values are reported for PSMs and unique peptides at $q \leq 0.01$. Confidence intervals are 95% paired confidence intervals calculated using Student's t distribution with $df = n - 1$. Paired p-values are reported as descriptive statistics and should be interpreted cautiously because the number of replicate acquisitions is small.

**Table A**

| Condition | Level | Comparator | n | ProjDIA mean ± SD | Comparator mean ± SD | Mean paired difference | Relative difference (%) | 95% CI | Paired p |
|---|---|---|---|---|---|---|---|---|---|
| 0.125 µg | PSM | CHIMERYS | 2 | 5180.5 ± 72.8 | 4926.5 ± 21.9 | +254.0 | +5.2 | [−597.3, +1105.3] | 0.164 |
| 0.125 µg | PSM | CsoDIAq | 2 | 5180.5 ± 72.8 | 3929.5 ± 0.7 | +1251.0 | +31.8 | [+603.0, +1899.0] | 0.026 |
| 0.125 µg | peptide (unique) | CHIMERYS | 2 | 3727.5 ± 116.7 | 3086.5 ± 38.9 | +641.0 | +20.8 | [−756.7, +2038.7] | 0.108 |
| 0.125 µg | peptide (unique) | CsoDIAq | 2 | 3727.5 ± 116.7 | 2784.5 ± 88.4 | +943.0 | +33.9 | [+688.9, +1197.1] | 0.013 |
| 0.25 µg | PSM | CHIMERYS | 2 | 6949.0 ± 12.7 | 6535.5 ± 111.0 | +413.5 | +6.3 | [−469.6, +1296.6] | 0.106 |
| 0.25 µg | PSM | CsoDIAq | 2 | 6949.0 ± 12.7 | 5177.0 ± 48.1 | +1772.0 | +34.2 | [+1225.6, +2318.4] | 0.015 |
| 0.25 µg | peptide (unique) | CHIMERYS | 2 | 4899.0 ± 73.5 | 4021.5 ± 81.3 | +877.5 | +21.8 | [−513.8, +2268.8] | 0.079 |
| 0.25 µg | peptide (unique) | CsoDIAq | 2 | 4899.0 ± 73.5 | 3519.5 ± 31.8 | +1379.5 | +39.2 | [+432.9, +2326.1] | 0.034 |
| 0.5 µg | PSM | CHIMERYS | 2 | 6615.5 ± 57.3 | 6585.5 ± 48.8 | +30.0 | +0.5 | [−46.2, +106.2] | 0.126 |
| 0.5 µg | PSM | CsoDIAq | 2 | 6615.5 ± 57.3 | 5206.5 ± 190.2 | +1409.0 | +27.1 | [+214.6, +2603.4] | 0.042 |
| 0.5 µg | peptide (unique) | CHIMERYS | 2 | 4736.5 ± 3.5 | 4016.5 ± 57.3 | +720.0 | +17.9 | [+173.6, +1266.4] | 0.038 |
| 0.5 µg | peptide (unique) | CsoDIAq | 2 | 4736.5 ± 3.5 | 3496.0 ± 229.1 | +1240.5 | +35.5 | [−849.7, +3330.7] | 0.084 |
| 1 µg | PSM | CHIMERYS | 2 | 7034.0 ± 80.6 | 6610.0 ± 189.5 | +424.0 | +6.4 | [−554.4, +1402.4] | 0.114 |
| 1 µg | PSM | CsoDIAq | 2 | 7034.0 ± 80.6 | 5048.0 ± 79.2 | +1986.0 | +39.3 | [+550.2, +3421.8] | 0.036 |
| 1 µg | peptide (unique) | CHIMERYS | 2 | 5047.0 ± 140.0 | 4130.0 ± 67.9 | +917.0 | +22.2 | [+269.0, +1565.0] | 0.035 |

| | | | | | | | | | |
|---|---|---|---|---|---|---|---|---|---|
| 1 µg | peptide (unique) | CsoDIAq | 2 | 5047.0 ± 140.0 | 3500.0 ± 66.5 | +1547.0 | +44.2 | [−308.1, +3402.1] | 0.060 |

**Table B**

| Condition | Level | Comparator | n | ProjDIA mean ± SD | Comparator mean ± SD | Mean paired difference | Relative difference (%) | 95% CI | Paired p |
|---|---|---|---|---|---|---|---|---|---|
| 30K | PSM | CHIMERYS | 2 | 3978.5 ± 208.6 | 5874.0 ± 198.0 | −1895.5 | −32.3 | [−5548.5, +1757.5] | 0.096 |
| 30K | PSM | CsoDIAq | 2 | 3978.5 ± 208.6 | 4020.0 ± 140.0 | −41.5 | −1.0 | [−657.8, +574.8] | 0.549 |
| 30K | peptide (unique) | CHIMERYS | 2 | 1311.0 ± 145.7 | 1575.5 ± 13.4 | −264.5 | −16.8 | [−1452.5, +923.5] | 0.216 |
| 30K | peptide (unique) | CsoDIAq | 2 | 1311.0 ± 145.7 | 1205.0 ± 87.7 | +106.0 | +8.8 | [−415.0, +627.0] | 0.235 |
| 45K | PSM | CHIMERYS | 2 | 5191.0 ± 113.1 | 6112.5 ± 77.1 | −921.5 | −15.1 | [−1245.5, −597.5] | 0.018 |
| 45K | PSM | CsoDIAq | 2 | 5191.0 ± 113.1 | 4423.0 ± 275.8 | +768.0 | +17.4 | [−693.2, +2229.2] | 0.095 |
| 45K | peptide (unique) | CHIMERYS | 2 | 2376.5 ± 16.3 | 2321.5 ± 17.7 | +55.0 | +2.4 | [−249.9, +359.9] | 0.262 |
| 45K | peptide (unique) | CsoDIAq | 2 | 2376.5 ± 16.3 | 1957.0 ± 19.8 | +419.5 | +21.4 | [+95.5, +743.5] | 0.039 |
| 60K | PSM | CHIMERYS | 2 | 5847.5 ± 669.6 | 5984.5 ± 553.7 | −137.0 | −2.3 | [−1178.9, +904.9] | 0.343 |
| 60K | PSM | CsoDIAq | 2 | 5847.5 ± 669.6 | 4682.0 ± 575.6 | +1165.5 | +24.9 | [+320.5, +2010.5] | 0.036 |
| 60K | peptide (unique) | CHIMERYS | 2 | 2682.5 ± 222.7 | 2474.0 ± 195.2 | +208.5 | +8.4 | [−39.3, +456.3] | 0.059 |
| 60K | peptide (unique) | CsoDIAq | 2 | 2682.5 ± 222.7 | 2224.0 ± 148.5 | +458.5 | +20.6 | [−208.6, +1125.6] | 0.073 |
| 120K | PSM | CHIMERYS | 3 | 6740.0 ± 219.4 | 6545.0 ± 78.2 | +195.0 | +3.0 | [−515.1, +905.1] | 0.359 |
| 120K | PSM | CsoDIAq | 3 | 6740.0 ± 219.4 | 5192.7 ± 136.6 | +1547.3 | +29.8 | [+908.0, +2186.7] | 0.009 |
| 120K | peptide (unique) | CHIMERYS | 3 | 4792.3 ± 96.7 | 3939.3 ± 40.5 | +853.0 | +21.7 | [+570.6, +1135.4] | 0.006 |
| 120K | peptide (unique) | CsoDIAq | 3 | 4792.3 ± 96.7 | 3538.7 ± 178.1 | +1253.7 | +35.4 | [+841.1, +1666.2] | 0.006 |
| 240K | PSM | CHIMERYS | 3 | 6907.7 ± 132.2 | 6359.7 ± 39.7 | +548.0 | +8.6 | [+121.4, +974.6] | 0.031 |
| 240K | PSM | CsoDIAq | 3 | 6907.7 ± 132.2 | 4907.3 ± 220.0 | +2000.3 | +40.8 | [+1642.4, +2358.3] | 0.002 |

| | | | | | | | | | |
|---|---|---|---|---|---|---|---|---|---|
| 240K | peptide (unique) | CHIMERYS | 3 | 6640.7 ± 134.8 | 5056.7 ± 13.6 | +1584.0 | +31.3 | [+1281.9, +1886.1] | 0.002 |
| 240K | peptide (unique) | CsoDIAq | 3 | 6640.7 ± 134.8 | 4687.3 ± 157.2 | +1953.3 | +41.7 | [+1740.7, +2165.9] | 0.001 |

**Table S5. Fragment-level diagnostic summary for representative benchmark subsets.**

| Subset | Tool | Accepted target PSMs | Matched fragments per PSM, median (IQR) [range] | Missing expected fragments per PSM, median (IQR) [range] |
|---|---|---|---|---|
| 0.5 µg / 240k | ProjDIA | 20,723 | 6 (5–8) [3–10] | 4 (2–5) [0–7] |
| 0.5 µg / 240k | CsoDIAq | 14,699 | 7 (6–8) [5–10] | 2 (1–3) [0–5] |
| 1 µg / 120k | ProjDIA | 14,068 | 5 (4–7) [3–10] | 5 (3–6) [0–7] |
| 1 µg / 120k | CsoDIAq | 10,096 | 7 (5–8) [4–10] | 3 (2–4) [0–6] |

Fragment-level metrics were calculated for accepted target PSMs at $q \leq 0.01$. For CsoDIAq, expected fragments were taken from the reported Peaks(Library) field. For ProjDIA, the expected-fragment count was approximated using the benchmark setting of up to 10 retained library fragments per precursor because an explicit expected-fragment column was not present in the PSM-level output.

**Table S6. ProjDIA fragment-sharing multiplicity within isolation windows.**

| Subset | Observed peaks assessed | 1 peptide | 2 peptides | 3 peptides | ≥4 peptides | ≥2 peptides total |
|---|---|---|---|---|---|---|
| 0.5 µg / 240k | 129,953 | 127,168 (97.86%) | 2,669 (2.05%) | 109 (0.08%) | 7 (0.005%) | 2,785 (2.14%) |
| 1 µg / 120k | 79,785 | 78,223 (98.04%) | 1,523 (1.91%) | 37 (0.05%) | 2 (0.003%) | 1,562 (1.96%) |

Observed peaks were grouped by scan/query index and rounded observed m/z. Values indicate how many accepted peptide candidates were compatible with the same observed peak. Shared observed fragments are expected in chimeric DI-SPA spectra and were quantified here as a diagnostic of fragment-sharing ambiguity.

**Table S7. Species-entrapment search-space sizes, accepted peptide counts, observed entrapment rates, and entrapment-based FDP estimates.**

**Table S7A. Search-space definitions used for entrapment-based FDP estimates.**

| Analysis | Workflow(s) | Res. | Design / classification | N target | N entrapment | Shared excluded | r | 1+1/r |
|---|---|---|---|---|---|---|---|---|
| 10-run spectral-library species-entrapment | ProjDIA; CsoDIAq | - | Human/sample target; E. coli + yeast entrapment; artificial decoys excluded | 16,623 | 27,171 | 19 | 1.635 | 1.612 |
| ProjDIA paired synthetic/reference-entrapment holdout control | ProjDIA | 30K-240K | Human-only paired target/TRAP library; target and TRAP partitions matched one-to-one | 86,725 | 86,725 | N/A, paired mirror design | 1.000 | 2.000 |
| CHIMERYS post-search species-entrapment analysis | CHIMERYS | 30K-240K | Accepted peptides assigned post-search to in-silico human vs E. coli/yeast proteomes | 2,102,616 | 805,580 | 2,214 | 0.383 | 3.610 |

**Table S7B. ProjDIA/CsoDIAq direct comparison and ProjDIA paired holdout-control results.**

| Analysis | Workflow | Res. | Accepted target T | Accepted entrapment E | Observed entrapment rate E/(T+E) | Entrapment-based FDP estimate |
|---|---|---|---|---|---|---|
| 10-run spectral library | ProjDIA | - | 2,712 | 23 | 0.8410% | 1.3554% |
| 10-run spectral library | CsoDIAq | - | 1,134 | 9 | 0.7874% | 1.2691% |
| Paired holdout control | ProjDIA | 30K | 2,203 | 6 | 0.2716% | 0.5432% |
| Paired holdout control | ProjDIA | 45K | 4,600 | 9 | 0.1953% | 0.3905% |
| Paired holdout control | ProjDIA | 60K | 5,782 | 20 | 0.3447% | 0.6894% |
| Paired holdout control | ProjDIA | 120K | 10,279 | 21 | 0.2039% | 0.4078% |
| Paired holdout control | ProjDIA | 240K | 13,481 | 35 | 0.2590% | 0.5179% |

**Table S7C. CHIMERYS post-search species-entrapment results.**

| Analysis | Workflow | Res. | Accepted target T | Accepted entrapment E | Observed entrapment rate E/(T+E) | Entrapment-based FDP estimate |
|---|---|---|---|---|---|---|
| CHIMERYS species-entrapment | CHIMERYS | 30K | 1,562 | 17 | 1.0766% | 3.8867% |
| CHIMERYS species-entrapment | CHIMERYS | 45K | 2,330 | 19 | 0.8089% | 2.9200% |
| CHIMERYS species-entrapment | CHIMERYS | 60K | 2,785 | 15 | 0.5357% | 1.9340% |
| CHIMERYS species-entrapment | CHIMERYS | 120K | 4,135 | 22 | 0.5292% | 1.9105% |
| CHIMERYS species-entrapment | CHIMERYS | 240K | 5,113 | 15 | 0.2925% | 1.0560% |

Note: T and E are accepted non-decoy target and entrapment peptide counts at $q \leq 0.01$. The observed entrapment rate is E/(T+E). The entrapment-based FDP estimate is E(1+1/r)/(T+E), where $r = N_{entrapment}/N_{target}$. For the ProjDIA paired synthetic/reference-entrapment holdout control, the target and TRAP partitions were matched one-to-one, so $N_{target} = N_{entrapment} = 86{,}725$, r = 1, and the FDP estimate was calculated as 2E/(T+E). Shared-sequence exclusion is not applicable to the paired holdout-control design because sequence identity between target and TRAP entries is intentional. In this control, entrapment-origin entries remained searchable and reportable but were held out from mass-error recalibration and semi-supervised rescoring model fitting. For CHIMERYS, accepted peptide sequences were classified after searching by post-search species assignment to in-silico human, E. coli, and yeast proteomes; this analysis is a workflow-level species-entrapment analysis of accepted outputs, not an audit of CHIMERYS internal FDR estimation.

**Table S8. Recalibration and rescoring safeguards against double-dipping.**

| Analysis / condition | Recalibration and rescoring safeguard | Final score used | Observed entrapment rate E/(T+E) | Entrapment-based FDP estimate | Interpretation |
|---|---|---|---|---|---|
| Original / reproduced 120K behavior | Reference behavior; entrapment-origin entries were not excluded from fitting | Held-out fold logit score | 23 / 10,533 = 0.2184% | 0.4367% (reference only) | Reproduces the original source behavior and provides a reference point for the holdout control. |
| Entrapment holdout control 30K | Entrapment-origin entries excluded from recalibration and ML fitting | Base score | 6 / 2,209 = 0.2716% | 0.5432% | ML rescoring was skipped after holdout exclusion reduced the eligible-decoy count below the rescoring guard; q-values use the base score. |
| Entrapment holdout control 45K | Entrapment-origin entries excluded from recalibration and ML fitting | Base score | 9 / 4,609 = 0.1953% | 0.3905% | ML rescoring was skipped after holdout exclusion reduced the eligible-decoy count below the rescoring guard; q-values use the base score. |
| Entrapment holdout control 60K | Entrapment-origin entries excluded from recalibration and ML fitting | Base score | 20 / 5,802 = 0.3447% | 0.6894% | ML rescoring was skipped after holdout exclusion reduced the eligible-decoy count below the rescoring guard; q-values use the base score. |
| Entrapment holdout | Entrapment-origin entries excluded from | Held-out fold | 21 / 10,300 = 0.2039% | 0.4078% | Held-out fold scores were used. The controlled 120K comparison showed |

| | | | | | |
|---|---|---|---|---|---|
| control 120K | recalibration and ML fitting | logit score | | | that excluding entrapment-origin entries from fitting did not increase the entrapment rate or FDP estimate. |
| Entrapment holdout control 240K | Entrapment-origin entries excluded from recalibration and ML fitting | Held-out fold logit score | 35 / 13,516 = 0.2590% | 0.5179% | Held-out fold scores were used; entrapment-origin entries were excluded from recalibration and ML fitting. |

Note: Recalibration is a run-specific mass-error correction estimated from high-confidence target matches, not a flexible classifier. For the entrapment holdout-control rows, entrapment-origin entries remained searchable and reportable but were excluded from mass-error recalibration fitting and from semi-supervised rescoring model fitting. When ML rescoring was enabled, ProjDIA used scan-grouped held-out cross-validation, so each scan group received a score from a model not trained on that scan group. When excluding entrapment-derived decoys reduced the eligible-decoy count below the rescoring guard, ML rescoring was skipped and the base score was retained. For the paired synthetic/reference-entrapment library, $N_{target} = N_{entrapment} = 86{,}725$, $r = 1$, and the entrapment-based FDP estimate was calculated as $2E/(T+E)$. Peptide-level results are reported at $q \leq 0.01$.

**Table S9. Protein-level entrapment sensitivity in the ten-run species-entrapment analysis**.

Protein groups were inferred from peptides passing $q \leq 0.01$; therefore, these values are observed protein-level entrapment diagnostics, not formal protein-level FDR estimates.

| Protein support rule | Target protein groups | Entrapment protein groups | Total protein groups | Observed protein-level entrapment fraction | Entrapment protein groups supported by one peptide |
|---|---|---|---|---|---|
| As reported: peptide $q \leq 0.01$ followed by protein inference | 1,030 | 22 | 1,052 | 2.09% | 21 / 22 |
| ≥1 unique peptide | 954 | 15 | 969 | 1.55% | 15 / 15 |
| ≥2 unique peptides | 565 | 0 | 565 | 0.00% | 0 / 0 |
| ≥2 peptides total | 682 | 1 | 683 | 0.15% | 0 / 1 |

Notes: The 2.09% value is an observed protein-level entrapment fraction among peptide-level-filtered protein groups, not a formal protein-level FDR estimate. The sensitivity analysis shows that observed protein-level entrapment is concentrated in single-peptide protein groups. Requiring ≥2 supporting peptides reduces the observed protein-level entrapment fraction to 0.15% using ≥2 peptides total or 0.00% using ≥2 unique peptides.

**Table S10. Comparison of ProjDIA and CsoDIAq LFQ ratio accuracy for the three-species mixture under matched post-processing filters**.

ProjDIA and CsoDIAq were evaluated using the same raw files, spectral library, mass tolerance, MaxLFQ quantification, and downstream filtering criteria. Expected $\log_2(A/B)$ ratios were yeast = +1, human = 0, and E. coli = −2. Ratio error was calculated as observed $\log_2(A/B)$ minus the expected $\log_2(A/B)$ ratio. The yeast-to-E. coli spread summarizes the observed separation between the enriched and depleted species; the expected spread is 3.0.

| Metric | ProjDIA | CsoDIAq |
|---|---|---|
| Median log2(A/B) ratio error, yeast; expected = +1 | +0.211 | −0.200 |
| Median log2(A/B) ratio error, human; expected = 0 | +0.139 | +0.109 |
| Median log2(A/B) ratio error, E. coli; expected = −2 | −0.067 | +0.489 |
| Median absolute log2(A/B) ratio error, peptide level | 0.166 | 0.197 |
| Median absolute log2(A/B) ratio error, protein level (MaxLFQ) | 0.148 | 0.215 |
| Yeast-to-E. coli log2 ratio spread, peptide level; expected = 3.0 | 3.28 | 2.31 |
| Yeast-to-E. coli log2 ratio spread, protein level (MaxLFQ); expected = 3.0 | 3.36 | 1.19 |

The small positive ratio error in the human 1:1 channel was observed for both workflows, consistent with a shared sample-level or normalization component. ProjDIA preserved the expected yeast-to-E. coli separation more closely than CsoDIAq under these matched conditions, indicating less ratio compression in this three-species LFQ comparison.

**Table S11. CHIMERYS output audit for the MitoTox dataset.**

| Audit item | Log result | Interpretation |
|---|---|---|
| Raw-file IDs processed | 132 | Raw files were processed by the CHIMERYS workflow. |
| Instrument reported in log | Orbitrap Fusion Lumos | Raw-file metadata were recognized. |
| Preliminary target PSMs at 1% FDR per raw-file entry | Median 106; range 7–328 | Preliminary search statistics were generated. |
| Preliminary target peptides at 1% FDR per raw-file entry | Median 76; range 7–245 | Preliminary peptide-level statistics were generated. |
| Final FDR input | 2264 total PSMs; 2245 target PSMs; 19 decoy PSMs | A final global FDR input table was generated. |
| Final FDR status | Fold 3 had no PSMs below eval_fdr = 0.001; FDR calculation failed | Final global confidence estimation did not complete under the tested settings. |
| Final comparable $q \leq 0.01$ PSM/protein table | Not available | CHIMERYS could not be carried forward into the same downstream MitoTox protein-level analysis. |
